# Immunological responses and gut microbial shift of fall armyworm *Spodoptera frugiperda* challenged with entomopathogens

**DOI:** 10.3389/fmicb.2026.1929843

**Published:** 2026-09-14

**Authors:** Martha W. Nderitu, Fathiya M. Khamis, Inusa J. Ajene, Steven G. Nyanjom, Komivi S. Akutse

**Affiliations:** 1 International Centre of Insect Physiology and Ecology (icipe), Nairobi, Kenya; 2 Department of Biochemistry, Jomo Kenyatta University of Agriculture and Technology (JKUAT), Nairobi, Kenya; 3 Department of Zoology and Entomology, University of Pretoria, Hatfield, South Africa; 4 Unit for Environmental Sciences and Management, North-West University, Potchefstroom, South Africa

**Keywords:** *Bacillus thuringiensis*, fall armyworm, hemocytes, *Metarhizium anisopliae*, microbiome, mortality

## Abstract

Fall armyworm (FAW), *Spodoptera frugiperda* is a dangerous lepidopteran pest that threatens maize production globally. Biopesticides such as entomopathogenic fungi (EPF) and *Bacillus thuringiensis* present safer alternatives to chemical pesticides for FAW management. This study investigated the impact of *Metarhizium anisopliae* and *B. thuringiensis* on the mortality, cellular immune response, and microbiome of FAW under different temperature regimes (15 °C, 20 °C, 25 °C, 30 °C, and 35 °C). *M. anisopliae* isolates ICIPE 7 and ICIPE 78 caused higher FAW larval mortality than *B. thuringiensis* isolates Bt43 and HD263 across all temperatures. *M. anisopliae* infection caused a more notable reduction in total hemocyte count (THC) in FAW, in contrast with *B. thuringiensis* infection, compared to control FAW groups. Plasmatocytes and granulocytes dominated the differential hemocyte counts in both challenged and control FAW larvae. Microbiome analysis of entomopathogen-infected FAW showed marginal changes in the microbiome diversity, with larvae challenged with ICIPE 7 and Bt43 having higher bacterial species richness than control samples assessed on days 0 and 10 post-exposure. Hence, we detected that infection with entomopathogens compromises the immune system of FAW and that *M. anisopliae* isolates ICIPE 7 and ICIPE 78 would be better candidates than *B. thuringiensis* isolates Bt43 and HD263 for integration into an IPM program against FAW.

## 1 Introduction

Fall armyworm (FAW) *Spodoptera frugiperda* (J.E. Smith), (Lepidoptera: Nuctuidae), is an important agricultural pest native to the tropical and subtropical regions of North, Central, and South America (Bateman et al., 2018). The pest was first confirmed in West and Central Africa in 2016 and thereafter reported in almost all countries in sub-Saharan Africa by 2017; ravaging cereal crops, especially maize, which is the staple food crop in the region (Akutse et al., 2019; Day et al., 2017). The pest therefore poses a serious threat to food security with an estimated loss in maize production observed between 4.1 and 17.7 million tons every year, resulting in losses valued at between US$ 1.088 and US$ 4.661 billion (Rwomushana et al., 2018). In Kenya, maize farmers lost an estimated 37% of their total crop to FAW in 2017 and 33% in 2018, which translates to loss of 1 million metric tons (De Groote et al., 2020).

To try and curb the pest spread and the associated losses, most maize farmers have relied heavily on chemical pesticides, despite some of these being classified as highly hazardous pesticides (HHP) with deleterious effects on the environment and human health (FAO, 2016, 2018). Furthermore, five commonly used insecticides against FAW (Cypermethrin, Deltamethrin, lambda-Cyhalothrin, Chlorpyrifos, and Permethrin) were found to persist in soil after application, posing negative effects on non-target soil-borne organisms (Togola et al., 2018). The tendency of FAW larvae to burrow into funnels and cobs, renders most traditional topical sprays ineffective, especially if there is delayed contact with the target pest (Bateman et al., 2021). The wrong usage of chemical pesticides has led to the development of pesticide resistance FAW populations and negative environmental impacts (Constantine et al., 2020; Rwomushana et al., 2018). Moreover, in countries like Brazil, farmers use up to five insecticide sprays per season to effectively combat FAW, which may be unaffordable for African smallholder farmers (Ribeiro et al., 2014).

Hence, there is a need for sustainable and eco-friendly alternatives, such as the use of entomopathogenic fungi (EPF), entomopathogenic bacteria (EPB), multiple nucleopolyhedroviruses, parasitoids, and predators (Abbas et al., 2022). Several entomopathogenic fungi isolates (*Metarhizium anisopliae* and *Beauveria bassiana*) have emerged as promising biological control agents against this destructive pest (Akutse et al., 2019) with the potential of effectively reducing FAW population. Key isolates identified to be effective against the immature stages of the pest include *M. anisopliae* ICIPE 7 and ICIPE 78 (Akutse et al., 2019). The incorporation of entomopathogenic fungi in FAW IPM is not only a sustainable and environmentally friendly solution but also offers unique advantages such as specificity to FAW, multiple modes of action, and potential for integration with other control options in IPM systems (Abbas et al., 2022; Inglis et al., 1995). Entomopathogenic bacteria (EPB) that have shown potential for the management of FAW populations in an IPM strategy include species in the *Bacillus* genus (*B. thuringiensis*) (Aarthi Helen et al., 2021; Abbas et al., 2022; Azazy et al., 2025; Polanczyk et al., 2000) and the *Streptomyces* genus (*S. hyderabadensis* and *S. xiamenenis*) (Aruna et al., 2023). EPB are highly specific to their hosts and once introduced to an ecosystem, they become a non-toxic part of the natural microflora, making them eco-friendlier than chemical pesticides (Kalha et al., 2014; Muralidharan et al., 2025). Hence, this study not only explored the efficacy of *M. anisopliae* and *B. thuringiensis* as FAW biocontrol agents (BCAs) but also documents the cellular immune responses and microbiome changes FAW undergoes in the process of epizootics.

Insects such as *S. frugiperda* have a powerful immune system based on innate and adaptive-like immunity to protect themselves from entomopathogenic bacteria, fungi, protozoa, nematodes, viruses, and parasites (Huot et al., 2019). Their cuticle acts as a physical barrier against biotic and abiotic factors and produces antimicrobial peptides (AMPs) upon wounding (Eleftherianos et al., 2013). This wounding can result from cuticle penetration due to pathogen invasion. Hence, both invasive immunity (focusing on the suppression of invading bacteria and eukaryotic pathogens) and innate immunity (which comprises humoral responses based on melanization and anti-microbial peptides) are triggered (Huot et al., 2019). Hemocytes (specialized phagocytic cells in insects) play significant roles in an insect’s response to infection (Senior and Titball, 2020). However, there is limited information on how these immune responses occur in FAW in response to EPF germination and mycosis or in response to lysis effects of Bt toxins in the gut. Moreover, the insect gut microbiome is not a passive participant in the host infection process but is involved in endosymbiosis, affecting various aspects of arthropod biology including nutrition, metabolism, stress resistance, behavior, body color, and immune responses (Duplouy and Hornett, 2018; Zug and Hammerstein, 2015). These endosymbionts can be primary or secondary, depending on whether they need their hosts to survive or only obtain non-critical benefits (Voirol et al., 2018).

Several studies have documented changes in insect gut microbiome, for instance, the infection of *Nilaparvata lugens* (Brown Planthopper) nymphs with *M. anisopliae* led to elevated gut bacterial load, decreased diversity, and changes in abundance, while in *Anopheles stephensi* (mosquito) adults, *Beauveria bassiana* caused an increase in gut bacterial loads and decreased diversity (Wang Z. et al., 2022). In addition, Abbas et al. (2022), Inglis et al. (1995), Maingi et al. (2023) also demonstrated that *M. anisopliae* isolates ICIPE 18 and ICIPE 20 elicited notable shifts in *Phthorimaea absoluta*’s gut microbiota, with *M. anisopliae* ICIPE 18 causing the highest mortality rate, while *M. anisopliae* ICIPE 20 exhibited the highest significant reduction in total hemocyte counts. The fungal infection further caused overgrowth and translocation of the opportunistic bacterium *Serratia marcescens* from the gut to the hemocoel, promoting insect death. Moreover, in *Lymantria dispar* (Gypsy moths), the elimination of the indigenous gut bacteria using antibiotics resulted in a reduction of larval mortalities when the moths ingested Bt toxins (Broderick et al., 2009). It was observed that the gut microflora was crucial for the expression of the Bt toxins, and their elimination negated the Bt toxins. In *Spodoptera exigua* (Beet armyworm) larvae, it was observed that the Bt toxin Cry1Ca disrupted the gut microbiota, increasing the bacterial load and decreasing diversity, which caused severe septicemia, with *Clostridium* and *Serratia* species becoming pathogenic, hence contributing to the larvae mortality (Li et al., 2022).

Despite pathogens like *M. anisopliae* and *B. thuringiensis* being excellent pest management solutions, little is known about FAW-EPF and FAW-Bt interactions. This study aimed to bridge the knowledge gap by generating crucial data on FAW-pathogen interactions and how both biotic and abiotic factors affect these BCAs’ effectiveness and their potential application.

## 2 Materials and methods

### 2.1 Subculture and suspension preparation of entomopathogenic fungal and bacterial isolates

*M. anisopliae* isolates ICIPE 7 and ICIPE 78 were obtained from *icipe*’s Arthropod Pathology Unit (APU) germplasm and subcultured on Saboroud Dextrose Agar (SDA) plates in complete darkness at 25 °C ± 2 °C for 15 days. The conidia were harvested from sporulated plates to prepare stock suspensions by flooding the plates with sterile distilled 0.05% Triton solution (Merck KGaA, Darmstadt, Germany) then scrapping with sterile scalpels into 15 mL universal bottles. Quantification to determine the concentration of the stock suspension was done with an improved Neubaeur hemocytometer (Paul Marienfeld GmbH & Co., KG, Lauda-Königshofen, Germany) under the Leica D500 light microscope at 40 × magnification (Leica Microsystems, Morrisville, NC, USA) (Horner et al., 1995) and subsequently diluted to a working concentration of 1 × 10^8^ conidia/mL for each isolate.

*B. thuringiensis* (Bt) isolates HD263 and Bt43 were also obtained from the *icipe*’s APU germplasm and were maintained on Luria-Bertani medium (LB) [10 g Tryptone, 5 g Yeast Extract, 15 g Technical Agar (Oxoid Ltd, Basingstoke, UK) and 10 g NaCl (Merck KGaA Darmstat, Germany)] in 90 mm Petri dishes then incubated at 37 °C for 24 h. Fresh cultures were mass produced in 250 mL flasks containing 50 mL sterile liquid LB broth (10 g Tryptone, 5 g Yeast Extract, 10 g NaCl in 1 L sterile distilled water) and placed in a New Brunswick Innova 44 incubator shaker (Eppendorf, Hamburg, Germany) set for 72 h at 37 °C and 150 rpm. The Bt cells were then transferred into sterile 50 mL Falcon tubes, pelleted at 3,900 rpm in the Eppendorf 5810R centrifuge (Eppendorf, Germany) for 10 min, rinsed with sterile water, centrifuged again; and then the pellet was resuspended in 10 mL sterile water to form stock suspensions. To quantify these cell suspensions, serial dilutions from 10^2^ to 10^8^ were done using 15 mL universal bottles containing 10 mL sterile distilled 0.05% Triton solution. Colony-forming units (CFUs) were determined by spread plating 100 μL of diluted suspensions onto LB plates and incubated at 37 °C for 18 h. The CFUs on the plates were then counted as a measure of live spores per 100 μL of corresponding dilute suspensions. The final concentration of Bt cells used for the bioassays was set at 1 × 10^8^ CFU/mL as described by Ochieng et al. (2024).

### 2.2 Rearing of fall armyworm

The 2*^nd^* instar FAW larvae used in this study were obtained from the *icipe* Animal Rearing and Quarantine Unit (ARQU). They were reared on fresh maize leaves (Akutse et al., 2019) at 23 °C, at a relative humidity of 60% and a photoperiod of 12 h light: 12 h dark. The initial colony of *S. frugiperda* was established using larvae collected on maize crops from Siaya and Homa Bay counties (−0.61401° Latitude, 34.09095° Longitude; 1215 m.a.s.l.), Kenya and reared for several generations prior to the experiments.

### 2.3 Exposure of FAW to *M. anisopliae* and *B. thuringiensis*

Bioassays for treating FAW larvae with *M. anisopliae* and *B. thuringiensis* isolates were conducted under controlled laboratory conditions using 2*^nd^* instar larvae. Each treatment comprised 30 larvae, replicated three times. Due to cannibalization, each larva was kept in a separate sterile 90 mm Petri dish.

Prior to each bioassay, spore viability tests were conducted by spread plating 3 × 10^6^ conidia/mL of each fungus onto Saboraud Dextrose Agar (SDA) (Oxoid Ltd; Basingstoke, UK) plates, incubating at 25 °C ± 2 °C, in complete darkness for 18 h. Growth was then terminated by staining culture plates with 1 mL lactophenol cotton blue, and the number of germinated spores against ungerminated spores was determined by counting them using a Leica D500 light microscope at 40 × magnification (Leica Microsystems, Morrisville, NC, USA). The spores were considered germinated when their germ tubes were at least twice the length of the spore’s diameter (Goettel and Inglis, 1997).

Fresh maize leaves were cut (4 cm × 3 cm); hereafter, a viable conidial suspension at the concentration of 1 × 10^8^ conidia/mL was sprayed onto the leaves on both sides, then left to air-dry. For control groups, the leaves were sprayed with sterile 0.05% Triton solution. The *M. anisopliae*-inoculated leaves were then transferred into 90 mm Petri dishes using sterile forceps, and a 2*^nd^* instar larva was randomly picked and placed into each Petri dish using a clean camel brush. Each treatment comprised 30 larvae, replicated three times; but due to cannibalization, each larva was kept in a separate Petri dish. The Petri dishes were sealed using parafilm to deter escapees, then placed in MIR-554 cooled incubators (PHC Europe B.V., Etten-Leur, Netherlands) set at specific temperatures (15 °C, 20 °C, 25 °C, 30 °C, and 35 °C). The same protocol was followed when setting bioassays involving treatment of FAW larvae with both isolates of *B. thuringiensis* (HD263 and Bt43) with viable spore solutions of 1 × 10^8^ CFU/mL. All the treatments were arranged in complete randomized design and replicated four times.

### 2.4 Mortality and cellular immunological response assessment

Mortality data was collected daily over a period of 10 days, post-pathogen exposure. In addition, samples for cellular responses of FAW (total and differential hemocyte counts) were also examined on days 0, 1, 5, and 10 post-pathogen inoculation under the different temperature regimes. On day 0, hemolymph was collected 2 h post-exposure of the larvae to both fungal and bacterial isolates. Hemolymph was collected from three live FAW larvae separately from both pathogen-infected and control groups, and the experiment was replicated three times (*n* = 9) as described by Gwokyalya et al. (2022) with slight modifications.

To obtain hemolymph, each larva was held between the thumb and index finger and pricked with a sterile lancet. For Total Hemocyte Count (THC), the insect’s oozing hemolymph was quickly collected with a sterile capillary tube, placed on a pre-cleaned microscope slide, and 1 μL collected for dilution with 9 μL of anticoagulant buffer (98 mM NaOH, 186 mM NaCl, 1.7 mM EDTA, and 41 mM citric acid; pH 4.5) in a 0.2 mL tube. The diluted hemolymph was loaded onto a new improved Neubauer chamber (Paul Marienfeld GmbH & Co., KG, Lauda-Königshofen, Germany) and cells were counted under the Leica D500 light microscope (Leica Microsystems) at 40 × magnification. Phenoloxidase (PO) activity was assessed by diluting 1 μL of hemolymph in 9 μL of phosphate-buffered saline (PBS), followed by incubation with 2 mg/mL Dihydroxyphenylalanine (L-DOPA) for 3 h. The change in color due to PO activity was then read every minute over 30 min at 490 nm using an Epoch 2 Microplate spectrophotometer on days 0, 1, 5, and 10 post-inoculation with *M. anisopliae* ICIPE 7 which was selected as the most virulent isolate and control at 25 °C (Supplementary Figure 7).

For differential hemocyte count (DHC), the insect’s hemolymph was placed on a clean microscope slide and then spread thinly along the slide with another slide. The hemolymph was left to dry at 25 °C, after which the slides were dipped in a 3:1 methanol: glacial acetic acid solution for fixing and left to air-dry. After fixing, the slides were dipped in a 5% Giemsa stain solution for 15 min, rinsed off with distilled water, then left to air-dry (Gwokyalya et al., 2022). The different kinds of hemocytes were then observed and counted under the Zeiss PrimoStar light Microscope (Zeiss Group, Baden-Württemberg, Germany), and the cell images were captured and their diameters measured using the Zeiss ZEN Blue software utilizing the AxioCam Camera (Zeiss Group). Hemocyte classification was done as described by Maingi et al. (2023).

### 2.5 Microbiome analysis

#### 2.5.1 DNA extraction and 16SrRNA metabarcoding

Fall armyworm larvae infected with *M. anisopliae* isolate ICIPE 7 and *B. thuringiensis* isolate Bt43 at 15 °C, 20 °C, and 25 °C were chosen for microbiome studies because ICIPE 7 emerged as the most potent *M. anisopliae* isolate while Bt43 is not only potent but also a new *B. thuringiensis* isolate with high potential against FAW and under investigation for commercialization. Due to high mortality, there were no surviving larvae for microbiome assessment at 30 °C and 35°C. Infected surviving FAW samples were collected on the 10*^th^* day post-pathogen exposure, to enable us to observe if there is any notable shift in the pest microbiome after treatment. Live samples from the control group (non-pathogen-infected larvae) were collected on both day 0 and day 10 post-exposure. Each treatment had five individuals, replicated three times (*n* = 15). Thereafter, genomic DNA extraction was done using the Isolate II Genomic DNA kit (Bioline, London, United Kingdom) as per the manufacturer’s protocol. DNA quantification and purity were confirmed using the Nanodrop 2000 (ThermoFischer Scientific, Wilmington, USA) spectrophotometer.

Sequencing library preparation was done using the SQK-16S024 16SrRNA Barcoding Kit [Oxford Nanopore Technologies (ONT) Inc., Oxford, UK] following the manufacturer’s protocol and which included a PCR step with the Eppendorf Mastercycler Nexus Gradient thermal cycler (Eppendorf, Germany). Each reaction was in a final volume of 50 μL comprising of 5x MyTaq Reaction Buffer (5 mM dNTPs, 15 mM MgCl_2_, Enhancer, and Stabilizer) (Bioline, UK), 0.625 μL of MyTaq DNA Polymerase (Bioline, UK), 10 μL of each 16S barcode, and 10 μL of each DNA template. The PCR cycling conditions were initial denaturation for 2 min at 95 °C, followed by 40 cycles of denaturation for 30 s at 95 °C, annealing for 45 s at 55 °C, extension for 1 min at 72 °C, and a final extension step of 10 min at 72 °C. The resulting amplicons were purified prior to sequencing using the Bioline Isolate II Nucleic Acid Purification Kit (Bioline, UK). The purified amplicons were then pooled and loaded into an ONT FLO-MIN106D (R9.4.1) flow cell; after which sequencing was performed using the MinION MK1C device (ONT) for 4 h. The MinKNOW software facilitated the MinION device to obtain raw data during the sequencing runs, while base calling was done using the Albacore Tool (v 2.3.4, ONT) on the ONT cloud. The resultant reads in FASTQ format were then uploaded onto the EPI2ME software to classify the reads and assign taxonomy using the WIMP Workflow^1^ while referencing the NCBI database.

### 2.6 Data analysis

Mortality data were adjusted using Abbott’s formula (Abbott, 1925) then subjected to comparative tests (Chi-square and one-way ANOVA/Pairwise MANOVA (*p* ≤ 0.05)) using the Generalized Linear Model (GLM) with quasi-binomial model to determine the effect of treatment and both temperature and treatment on larval mortality. Total hemocyte count (THC) data was also analyzed using comparative statistical tests using GLM (quasi-poisson procedure) to determine the difference in FAW THC caused by treatments and both treatment and temperature at days 0, 1, 5, and 10 post infection. Comparison among the treatments was done using Tukey’s HSD test at *p* ≤ 0.05 with R packages emmeans (Lenth, 2022) and multcomp (Horthon et al., 2008).

Differential hemocyte count analyses were performed using R Betareg package (Cribari-Neto and Zeileis, 2010) with beta regression to account for the beta-binomial data distribution (all treatments and control groups, days 0, 1, 5, and 10; and different hemocyte types) at 15 °C, 20 °C, 25 °C, and 30°C. THC and DHC at 30 °C were done up to day 5 due to high larval mortality caused by the treatment and temperature exposure. These analyses were carried out using R statistical software v 4.2.0 (R Core Team, 2022).

For microbiome data, a stacked bar graph was used to aid in visualization of taxa abundance in each sample, where taxa with reads below 0.30% collapsed and displayed as “others.” Comparative read count analysis was done at the genus level, while Alpha-diversity metrics were used to reveal species diversity within each sample. The metrics used in this study were Richness, Evenness, and Shannon diversity. Beta-diversity measures were done to show bacterial genera diversity across all samples and were calculated using the Jaccard dissimilarity index (Tang et al., 2021). To visualize the interpopulation distances, Principal Coordinate Analysis (PCoA) plots were generated. Both the Alpha and Beta diversity analyses were done using the “vegan” package (Oksanen et al., 2022) in R statistical software v4.1.2 (R Core Team, 2022).

## 3 Results

### 3.1 Effect of temperature on mortality on mortality of *S. frugiperda* inoculated with *M. anisopliae* and *B. thuringiensis*

The mortality data collected daily for 10 days post-infection showed that FAW larvae challenged with *M. anisopliae* ICIPE 7 consistently had the highest cumulative percentage mortalities at 15 °C (41.68 ± 1.01%; Figure 1A), 20 °C (35.05 ± 0.79%; Figure 1B), 25 °C (78.55 ± 2.95%; Figure 1C) and 30 °C (63.72 ± 1.94%; Figure 1D) compared to all other treatments. FAW larvae challenged with *M. anisopliae* ICIPE 78 had the second-highest cumulative mortalities at 15 °C (31.68 ± 0.85%), 20 °C (25.07 ± 0.52%), 25 °C (30.03 ± 0.72%); and 30 °C (42.02 ± 1.11%). On the other hand, *B. thuringiensis*-inoculated larvae had recorded extremely low mortalities at all temperatures, where sporadic larval deaths were recorded on some days. At 35 °C, 100% larval mortality was observed between days 1 and 2 post-challenge with both *M. anisopliae* and *B. thuringiensis* isolates.

**FIGURE 1:**
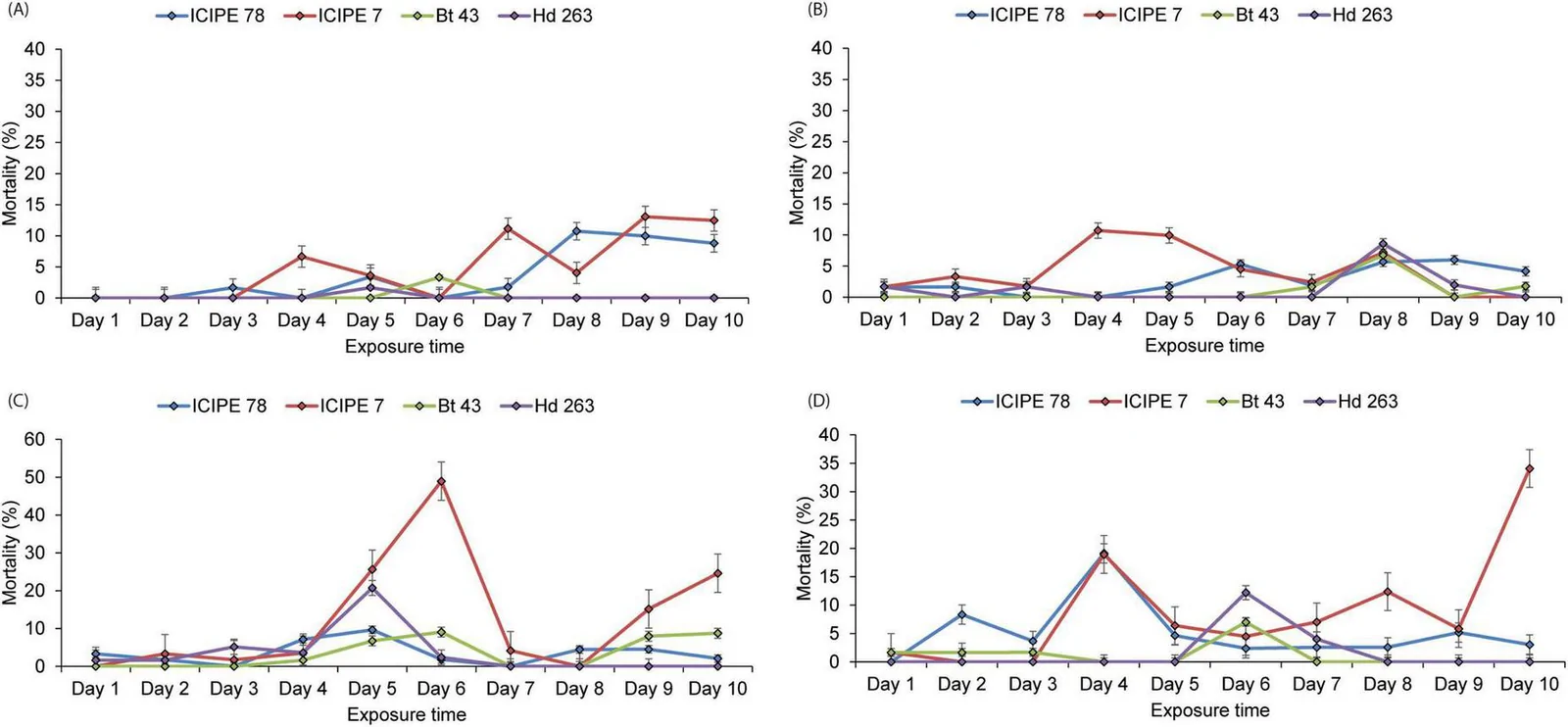
Daily percentage mortality of second instar *Spodoptera frugiperda* larvae challenged with *Metarhizium anisopliae* isolates ICIPE 78 and ICIPE 7, and *Bacillus thuringiensis* isolates Bt43 and HD263 at **(A)** 15 °C, **(B)** 20 °C, **(C)** 25 °C, and **(D)** 30° C.

A significant difference was noted between the Abbot-adjusted cumulative mortalities of FAW challenged larvae with *M. anisopliae* isolates ICIPE 7, ICIPE 78; and *B. thuringiensis* isolates Bt43 and HD263 at 15 °C (χ^2^ = 113.49, df = 3, *p* < 0.001), 20 °C (χ^2^ = 70.71, df = 3, *p* < 0.001), 25 °C (χ^2^ = 109.42, df = 3, *p* < 0.001), and 30 °C (χ^2^ = 132.15, df = 3, *p* < 0.001). Pairwise MANOVA revealed that both temperature and treatment had a significant effect on cumulative mortality rates among the treatments (χ^2^ = 84.83, df = 9, *p* < 0.001) (Figure 2).

**FIGURE 2:**
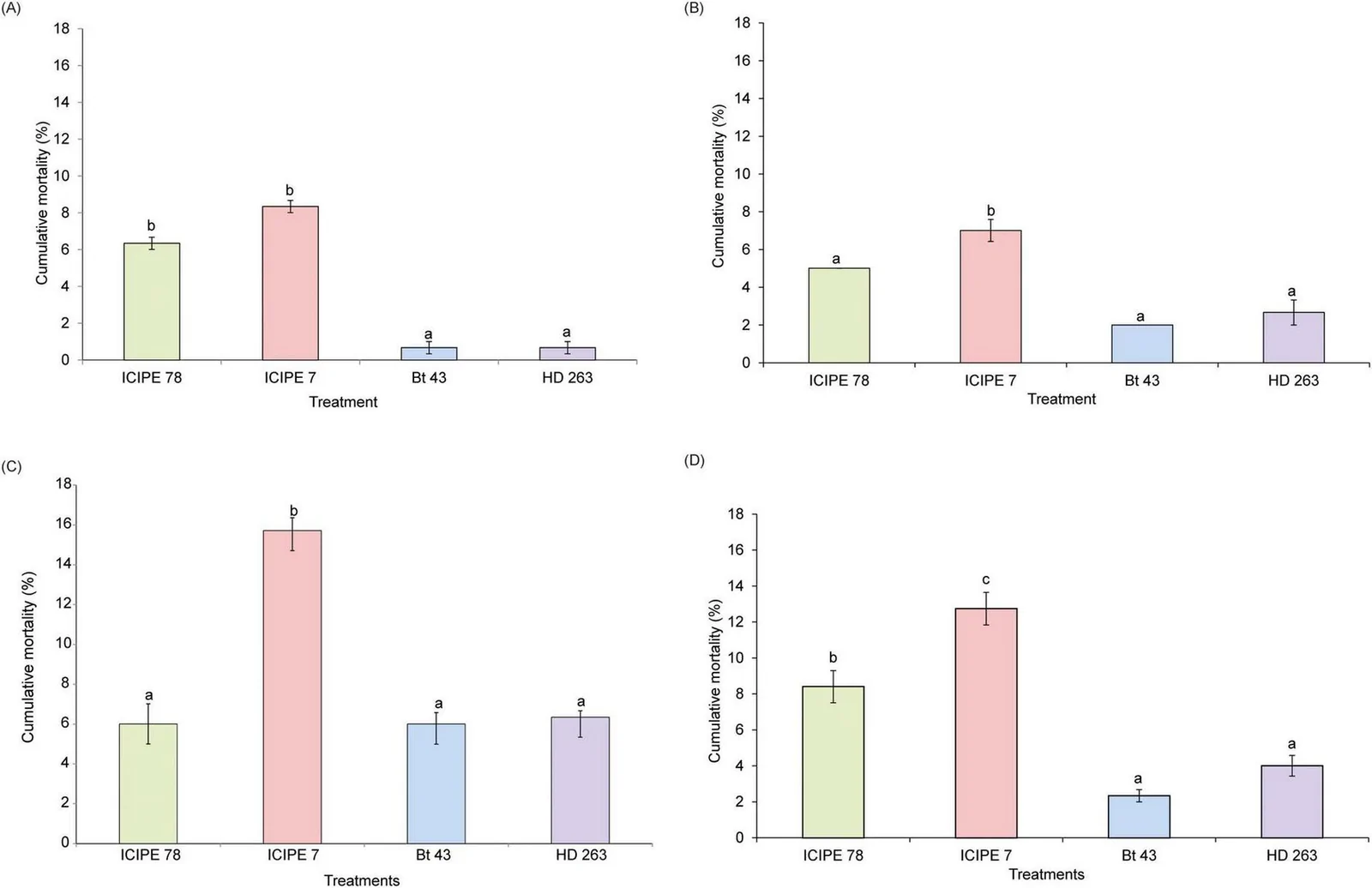
Cumulative mortality of second instar *Spodoptera frugiperda* challenged with *Metarhizium anisopliae* isolates ICIPE 78 and ICIPE 7 and *Bacillus thuringiensis* isolates Bt43 and HD263 at 15 °C **(A)**, 20 °C **(B)**, 25 °C **(C)**, and 30 °C **(D)**. The bars denote the means, and means followed by the same letters are not significantly different (at *P* < 0.05).

For the EPF, the highest cumulative mortality of FAW larvae challenged with *M. anisopliae* ICIPE 7 was observed at 25 °C (47.13 ± 0.41%) (Figure 2C) while the least was observed at 20 °C (21.03 ± 0.14%) (Figure 2B). The FAW larvae challenged with *M. anisopliae* ICIPE 78 had the highest cumulative mortality of 25.21 ± 0.19% at 30 °C (Figure 2D) and the lowest at 20 °C (15.04 ± 0.09%) (Figure 2B). For the *B. thuringiensis* isolates, FAW larvae challenged with Bt43 recorded the highest cumulative mortality at 25 °C (18 ± 0.14%) (Figure 2C) and the lowest mortality was observed at 15 °C (2 ± 0.05%) (Figure 2A). Similarly, the highest cumulative mortality of FAW larvae challenged with HD263 was observed at 25 °C (19.03 ± 0.21%) (Figure 2C), and the lowest at 15 °C (1 ± 0.03%) (Figure 2A).

### 3.2 Cellular immunological response

Entomopathogen-infected and the control treatments at 15 °C, 20 °C, 25 °C, and 30 °C were used for cellular immune response studies. This is because, the bioassays conducted at temperatures above 30 °C exhibited very high mortality rates after pathogen exposure, hampering hemolymph collection.

#### 3.2.1 Total hemocyte count

At 15 °C, there was no significant difference between the THC of FAW larvae challenged with *M. anisopliae* isolates ICIPE 7 and ICIPE 78 and control larvae (χ^2^ = 0.98, df = 2, *p* = 0.6127), and those challenged with *B. thuringiensis* isolates Bt43 and HD263 compared to control larvae (χ^2^ = 0.82, df = 2, *p* = 0.6623) on day 0. However, on days 1 and 5, significant differences in the THC of FAW challenged with *M. anisopliae* isolates compared to controls were observed (χ^2^ = 77.68, df = 2, *p* < 0.001; χ^2^ = 52.16, df = 2, *p* < 0.001; χ^2^ = 5.45, df = 2, *p* = 0.00135); while by at day 10, there was no significant difference noted between the THC of FAW inoculated with *M. anisopliae* isolates and controls (χ^2^ = 5.45, df = 2 *p* = 0.06558) (Figure 3A). For FAW challenged with the *B. thuringiensis* isolates, significant differences were observed between the THC of infected larvae compared to the controls on day 1 (χ^2^ = 35.91, df = 2, *p* > 0.001), day 5 (χ^2^ = 50.98, df = 2, *p* < 0.001), and day 10 (χ^2^ = 27.15, df = 2, *p* < 0.001) (Figure 3A).

**FIGURE 3:**
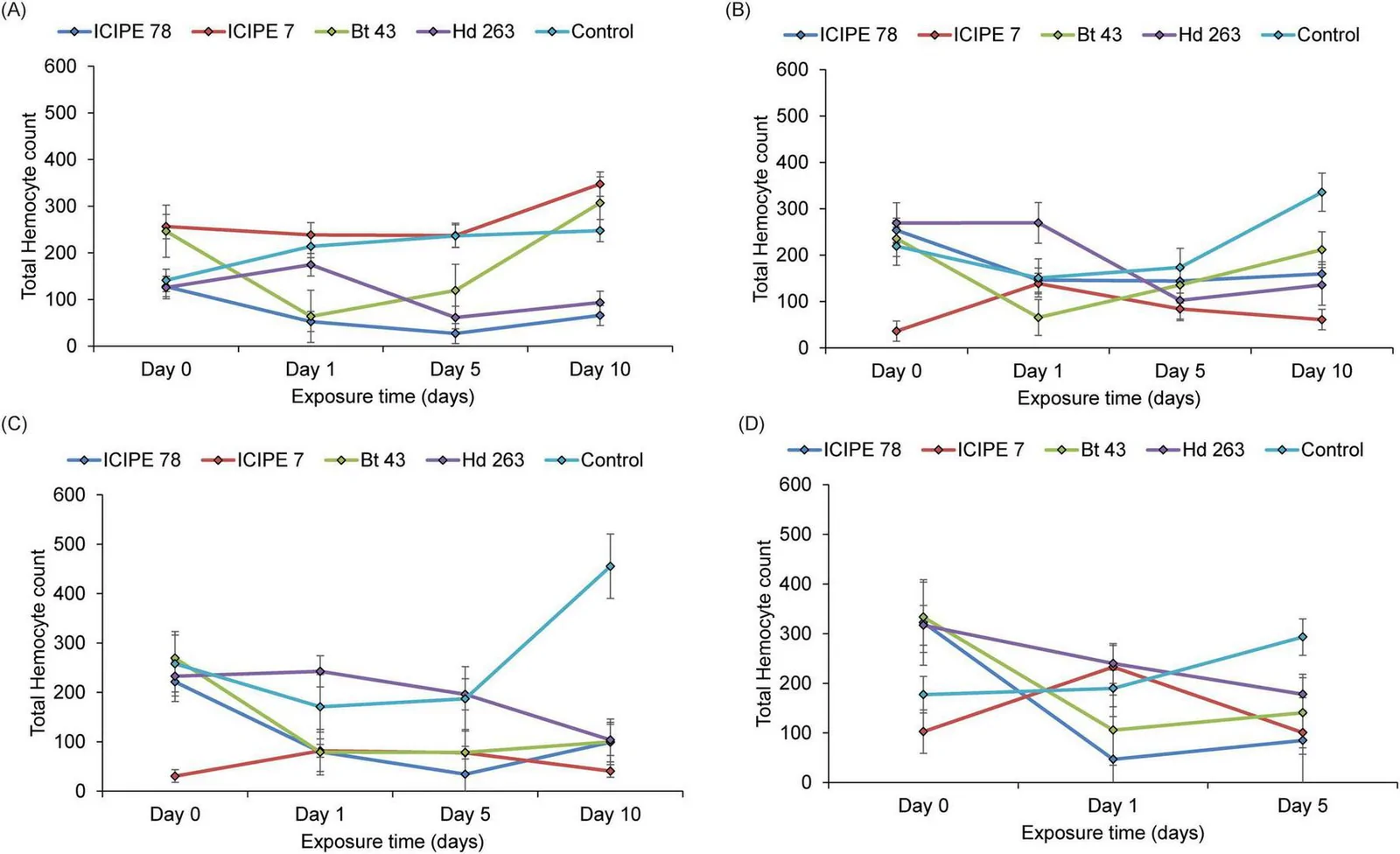
Effect of *Metarhizium anisopliae* isolates ICIPE 78 and ICIPE 7, and *Bacillus thuringiensis* isolates Bt43 and HD263, and temperature on total hemocyte count of *Spodoptera frugiperda* larvae at **(A)** 15 °C, **(B)** 20 °C, **(C)** 25 °C, and **(D)** 30 °C.

The THC of the FAW larvae challenged with *M. anisopliae* isolates ICIPE 7 and ICIPE 78, and those infected with the *B. thuringiensis* isolates Bt43 and HD263, declined notably compared to control larvae, especially between days 0 and 1. The THC of FAW infected with *M. anisopliae* isolate ICIPE 78 did not vary notably from days 1 to 5, while on day 10 larvae challenged with *M. anisopliae* isolate ICIPE 7 showed slight drop, then increased with time. The THC of FAW larvae challenged with *B. thuringiensis* isolates increased from day 1 to day 5 and then climbed notably by day 10 (Figure 3A).

At 20 °C, no significant difference was observed between the THC counts of FAW larvae challenged with *M anisopliae* isolates compared to control larvae (χ^2^ = 4.83, df = 2, *p* = 0.3052) and FAW larvae challenged with *B. thuringiensis* isolates versus control (χ^2^ = 4.53, df = 2, *p* = 0.1039) on day 0. However, significant differences were again noted in THC of FAW larvae infected with the *M. anisopliae* isolates versus control on day 1 (χ^2^ = 41.31, df = 2, *p* < 0.001), day 5 (χ^2^ = 72.39, df = 2, *p* < 0.001), and day 10 (χ^2^ = 9.09, df = 2, *p* = 0.0106). For the FAW larvae infected with the *B. thuringiensis* isolates, a significant difference in THC was observed between infected and control larvae on day 1 (χ^2^ = 44.89, df = 2, *p* < 0.001). On day 5, the difference between the THC of infected and control larvae was not significantly different (χ^2^ = 0.40, df = 2, *p* = 0.8206), while at day 10, a slightly significant variation was observed (χ^2^ = 6.32, df = 2, *p* = 0.04245). Furthermore, a drop in THC in larvae infected with both *M. anisopliae* isolates was noted, especially between days 0–1 and days 1–5, which further increased to similar levels of those observed in control larvae by day 10. For the larvae infected with *B. thuringiensis*, a slight increase in THC was observed from day 0 to day 1, after which THC remained constant up to day 5, then dropped at day 10 in larvae challenged with Bt43; and dropped on day 5, with an increase at day 10 in HD263-challenged larvae. The daily THC of *B. thuringiensis*-challenged larvae were more comparable to that of control larvae (Figure 3B).

At 25 °C, no significant differences in THC between the challenged and control groups for FAW larvae infected with *M. anisopliae* (χ^2^ = 0.82, df = 2, *p* = 0.6631) and *B. thuringiensis* (χ^2^ = 1.78, df = 2, *p* = 0.4102) were observed. For larvae infected with both *M. anisopliae* isolates, significant differences were observed between the THCs of challenged and control larvae groups on day 1 (χ^2^ = 48.55, df = 2 *p* < 0.001), day 5 (χ^2^ = 18.96, df = 2, *p* < 0.001), and day 10 (χ^2^ = 35.73, df = 2, *p* < 0.001). For larvae inoculated with the *B. thuringiensis* isolates, a similar trend was observed as compared with those examined at 20 °C where a significant difference was observed between the THC of infected and control larvae groups on day 1 (χ^2^ = 7.14, df = 2, *p* = 0.02823), but there were no variations observed on day 5 (χ^2^ = 1.77, df = 2, *p* = 0.4128), while at day 10, a slight significant variation between the THC of infected versus control larvae was noted (χ^2^ = 8.77, df = 2, *p* = 0.01248) (Figure 3C). Additionally, at 25 °C, the THCs of *M. anisopliae*-challenged FAW larvae dropped sharply from day 0 to 1 and remained low throughout until day 10 compared to control larvae. In contrast, those infected with both *B. thuringiensis* isolates had a notably higher THC compared to *M. anisopliae*-challenged larvae, and their THC was more comparable to that of the control larvae. The THC of Bt43-challenged larvae dropped slightly on day 1, then increased on days 5 and 10, while the THC of HD263-challenged larvae increased on day 1, dropped slightly on day 5, and then increased notably on day 10 (Figure 3C).

Finally, at 30 °C, there was no significant difference between the THC of FAW larvae inoculated with *M. anisopliae* (χ^2^ = 1.97, df = 2, *p* = 0.3745) and *B. thuringiensis* (χ^2^ = 0.87, df = 2, *p* = 0.6742) compared to control larvae. At day 1, there was a significant difference between the THC of larvae infected with *M. anisopliae* versus control (χ^2^ = 107.32, df = 2, *p* < 0.001) and the THC of larvae infected with *B. thuringiensis* isolates versus control (χ^2^ = 7.67, df = 2, *p* = 0.02161). At day 5, the THC of larvae infected with *M. anisopliae* was not significantly different from that of the control larvae (χ^2^ = 3.69, df = 2, *p* = 0.1581), but the THC of larvae infected with *B. thuringiensis* was observed to be significant compared to that of uninoculated larvae (χ^2^ = 70.98, df = 2, *p* < 0.001). The drop in THC of FAW larvae challenged with *M. anisopliae* isolates ICIPE 7 and ICIPE 78 at 30 °C was notable from days 0 to 1 just like with those examined at 25 °C and 20 °C; and remained low up to day 5. However, the THC of larvae challenged with *B. thuringiensis* isolates Bt43 and HD263 showed a slight drop from day 0 to day 1; while by day 5, the THC of Bt43-challenged larvae dropped notably in contrast to that of HD263-challenged larvae, which showed a minor drop Figure 3D). By day 10, larval mortality was high, so we were unable to obtain survival larvae to extract the hemolymph for examination.

#### 3.2.2 Differential hemocyte count

Various hemocyte types were observed in the hemolymph of EPF-infected, *B. thuringiensis*-challenged, and control FAW larvae. Plasmatocytes (PLs) were distinguished by their densely-stained nucleus and less-densely stained cytoplasm and measured about 25–28 μm. They were observed in clusters and as individual cells. The nucleus was centrally located in the cell, and both round and spindle-shaped plasmatocytes were observed (Supplementary Figure 1). Prohemocytes (PRs) looked like plasmatocytes but were distinguished by being notably smaller, measuring between 3.7 and 5.5 μm. Their nuclei were densely stained, while their cytoplasm showed little to no staining (Supplementary Figure 2). Granulocytes (GRs) were distinguished by the granules in their nucleus. Granulocytes’ nuclei were centrally located in the cell, with a less densely stained cytoplasm (Supplementary Figure 3). Like plasmatocytes, granulocytes were numerous and relatively large, measuring in at 25–30 μm. Spherulocytes (SPs) and Oenocytoids (OEs) were rare. Spherulocytes were distinguished by having massive spheres in their cytoplasm that appeared to conceal the nucleus and measured between 27 and 37 μm (Supplementary Figure 4). Oenocytoids looked like plasmatocytes but were distinguished by their oval shape and having their nucleus located on one end of the cell and were 25–28 μm in diameter (Supplementary Figure 5). Adipohemocytes (ADs) had undefined shapes, and their nuclei were difficult to distinguish, but the lipid-like inclusions in their cytoplasm set them apart, and they measured between 18 and 21 μm (Supplementary Figure 6).

Granulocytes were the most abundant cells, representing 45%–80% of the cells observed, followed closely by plasmatocytes with 30%–55%. Prohemocytes were constant in number and made up 5%–20% of the hemocytes observed. Oenocytoids, Spherulocytes, and Adipohemocytes were the least abundant, making up 0.1%–3% (Supplementary Table 1).

Bioassays done at 15 °C revealed a significant difference in the Granulocytes, Plasmatocytes, and Prohemocytes of FAW challenged with both *M. anisopliae* ICIPE 78 and ICIPE 7 and *B. thuringiensis* Bt43 and HD263 compared to control on day 0 (GR: χ^2^ = 246.5, z = 2.239, *p* = 0.0251; PL: χ^2^ = 1426.6, z = 2.237, *p* = 0.0253). On day 1, the number of Granulocytes was significantly higher in infected FAW larvae compared to control groups (χ^2^ = 1095.5, z = 2.237, *p* = 0.0253) while that of Plasmatocytes was significantly lower (χ^2^ = 417.3, z = 2.232, *p* = 0.0256). DHC analysis at day 5 post exposure also showed a significant difference in the number of Granulocytes and Plasmatocytes (GR: χ^2^ = 1131.5, z = 2.235, *p* = 0.0254; PL: χ^2^ = 949.8, z = 2.236, *p* = 0.0253). At day 10 post exposure, the number of Plasmatocytes and Granulocytes in infected FAW was much lower and more comparable to the control groups, but still significantly different with *M. anisopliae* ICIPE 7-challenged FAW larvae having a higher number of Granulocytes (GR: χ^2^ = 162.81, z = 2.24, *p* = 0.0251; PL: χ^2^ = 880.1, z = 2.236, *p* = 0.0253). DHC analysis results at Days 0, 1, and 5 post exposure to EPF and *B. thuringiensis* isolates at 15 °C were comparable, with notable changes in the numbers of each cell type being observed mostly at day 10 (Supplementary Table 1a).

Differential hemocyte count analysis of FAW larvae infected with EPF and *B. thuringiensis* isolates at 20 °C also revealed a significant difference in the number of Granulocytes and Plasmatocytes among infected and control groups (GR: χ^2^ = 95.07, z = 2.247, *p* = 0.0247; PL: χ^2^ = 99.71, z = 2.243, *p* = 0.0249). FAW larvae challenged with Bt43 and HD263 isolates had a higher number of Granulocytes and a lower number of Plasmatocytes compared to those challenged with *M. anisopliae* ICIPE 7 and ICIPE 78 as well as the control group. At day 1 post exposure, the number of Granulocytes in the *B. thuringiensis*-challenged FAW larvae dropped significantly but increased in the EPF-challenged larvae (χ^2^ = 424.2, z = 2.238, *p* = 0.0252) while the number of Plasmatocytes increased in the *B. thuringiensis*-challenged larvae and dropped in those that were infected with the EPF isolates (χ^2^ = 762.1, z = 2.237, *p* = 0.0253). At day 5, the number of Plasmatocytes and Granulocytes remained significantly different in the infected and control larvae groups (GR: χ^2^ = 338.5, z = 2.239, *p* = 0.0252; PL: χ^2^ = 307.5, z = 2.238, *p* = 0.0252). FAW larvae inoculated with *M. anisopliae* ICIPE 78 and control groups had a higher number of Plasmatocytes compared to Granulocytes, while groups challenged with *M. anisopliae* ICIPE 7 and the *B. thuringiensis* isolates recorded a higher number of Granulocytes than Plasmatocytes. On day 10 post exposure, FAW larvae infected with *M. anisopliae* ICIPE 78 and controls still had slightly more Plasmatocytes than Granulocytes, while the larvae infected with *M. anisopliae* ICIPE 7, Bt43, and HD263 maintained more Granulocytes than Plasmatocytes (GR: χ^2^ = 99.11, z = 2.243, *p* = 0.0247; PL: 35.00, z = 2.253, *p* = 0.0243). At 20 °C, a lower number of Adipohemocytes was observed on days 1 and 5 post exposure, then significantly increased on day 10 (day1: χ^2^ = 123.74, z = 2.236, *p* = 0.0254; day5: χ^2^ = 370.9, z = 2.237, *p* = 0.0253; day10: χ^2^ = 46.26, z = 2.248, *p* = 0.0246) (Supplementary Table 1b).

The assessment of DHC in FAW at 25 °C revealed a significant difference in the number of Granulocytes and Plasmatocytes on day 0 (GR: χ^2^ = 187.62, z = 2.241, *p* = 0.025; PL: χ^2^ = 158.90, z = 2.239, *p* = 0.0252), where there was a notable gap between the number of Granulocytes and Plasmatocytes across infected and control larvae. At day 1, the number of Granulocytes and Plasmatocytes remained significantly different (GR: χ^2^ = 380.9, z = 2.239, *p* = 0.0252; PL: χ^2^ = 1282.3, z = 2.237, *p* = 0.0253), where Granulocytes number had slightly dropped while that of Plasmatocytes had increased among infected larval groups. On both days 5 and 10 post exposure, the number of Granulocytes was significantly higher than that of Plasmatocytes among infected larvae, while the margin between the two cell types in the control group was narrower: (day 5: GR: χ^2^ = 516.6, z = 2.238, *p* = 0.0252; PL: χ^2^ = 600.3, z = 2.237, *p* = 0.0253; day 10: GR: χ^2^ = 646.5, z = 2.237, *p* = 0.0253; PL: χ^2^ = 604.3, z = 2.237, *p* = 0.0253). In addition, a notable number of Adipohemocytes were observed in both challenged and control FAW larvae on days 5 and 10 post-exposure, respectively: (χ^2^ = 74.73, z = 2.245, *p* = 0.0248; χ^2^ = 59.86, z = 2.25, *p* = 0.0244) (Supplementary Table 1c).

Finally, DHC analysis of FAW larvae challenged with EPF and *B. thuringiensis* at 30 °C also revealed a significantly higher number of Granulocytes than Plasmatocytes in infected and control larval groups (GR: χ^2^ = 162.31, z = 2.243, *p* = 0.0249; PL: χ^2^ = 273.2, z = 2.239, *p* = 0.0252). On day 1, the number of Granulocytes remained high while that of Plasmatocytes dropped across all FAW larval groups compared to day 0 (GR: χ^2^ = 842.2, z = 2.237, *p* = 0.0253; PL: χ^2^ = 342.9, z = 2.238, *p* = 0.0252). At day 5, the number of Plasmatocytes increased among the infected and control larvae relative to that of Granulocytes, which remained either constant or dropped (GR: χ^2^ = 397.7, z = 2.238, *p* = 0.0252; PL: χ^2^ = 951.8, z = 2.237, *p* = 0.0253). Adipohemocytes were also observed on days 1 and 5 post-exposure among infected and control groups (χ^2^ = 99.09, z = 2.24, *p* = 0.0251; χ^2^ = 38.81, z = 2.253, *p* = 0.0243) (Supplementary Table 1d).

Throughout our bioassays, the number of Prohemocytes remained relatively low, with slight fluctuations observed among the differently challenged FAW larvae and controls across time and changed with temperatures. Moreover, other than the consistently high numbers of Plasmatocytes and Granulocytes, and negligible numbers of Oenocytoids and Spherulocytes, no clear trend was noted in FAW larvae DHC resulting from treatment with *M. anisopliae* ICIPE 78 and ICIPE 7, and *B. thuringiensis* isolates Bt43 and HD263 at 15 °C, 20 °C, 25 °C, and 30 °C (Supplementary Table 1).

### 3.3 Microbiome analyses

The analyses of the microbiome revealed that Firmicutes (Bacillota) and Pseudomonadota, were the most abundant bacterial phyla. At the genus level, *Klebsiella*, *Serratia*, *Enterococcus*, *Wolbachia*, and *Escherichia* were the most abundant (Supplementary Figure 8). At 15 °C, the most abundant bacterial genus in the *M. anisopliae* ICIPE 7-challenged FAW larvae was *Klebsiella* (17.60%) while that in *B. thuringiensis* Bt43-challeged larvae was *Serratia* (24.17%) (Figure 4A). At 20 °C, the most abundant genus in ICIPE 7-challenged larvae was *Enterococcus* (31.46%) while in Bt43-challenged larvae was *Klebsiella* (20.06%) (Figure 4B). At 25 °C, the most abundant genus in FAW larvae challenged with ICIPE 7 was *Escherichia* (27.81%) while those challenged with Bt43 showed *Enterococcus* (43.82%) as the most abundant (Figure 4C).

**FIGURE 4:**
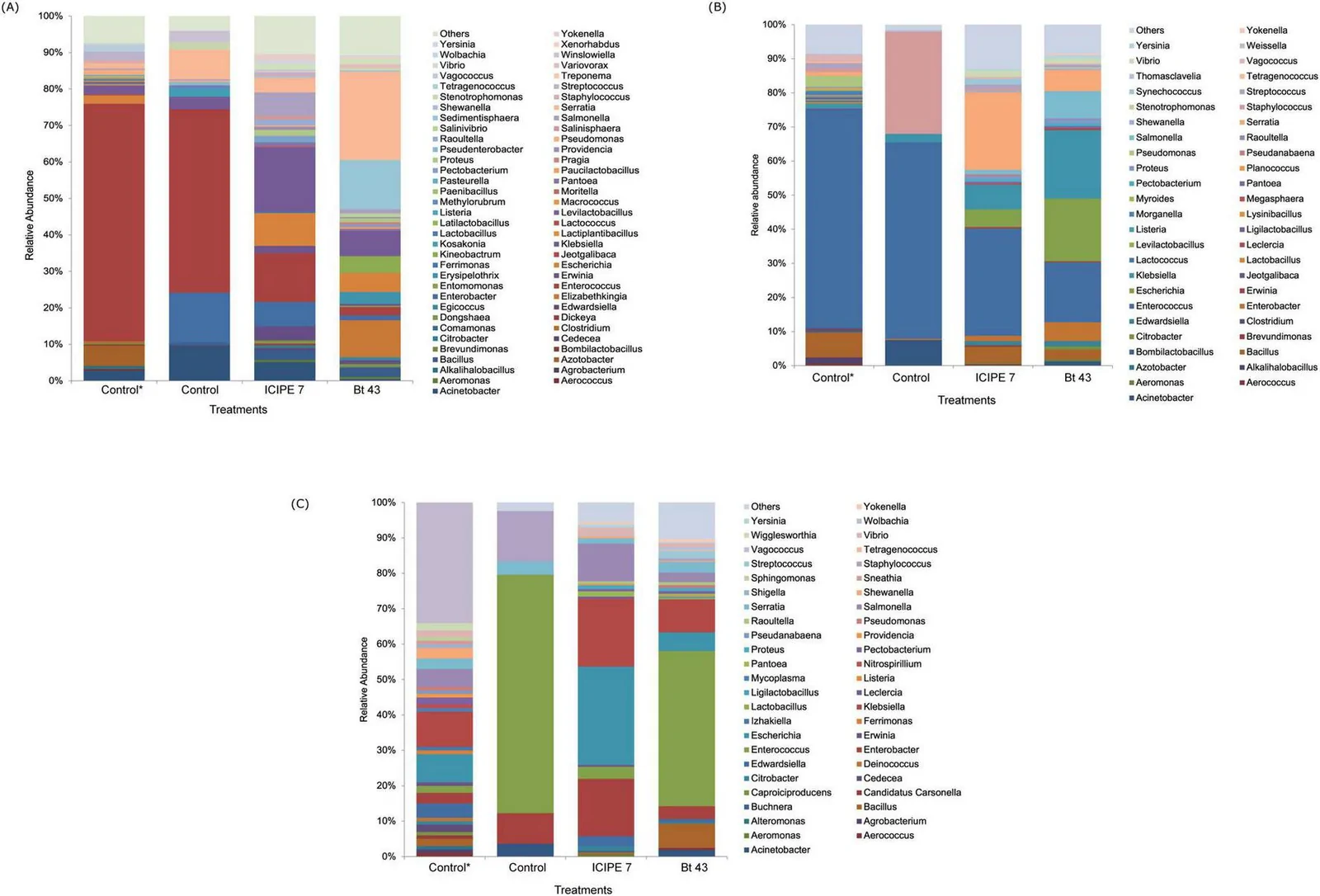
Relative abundance of bacterial communities in the gut of *Spodoptera frugiperda* larvae at genus level after infection with *Metarhizium anisopliae* isolate ICIPE 7 and *Bacillus thuringiensis* isolate Bt43 at 15 °C **(A)**, 20 °C **(B)**, and 25 °C **(C)**.

#### 3.3.1 Alpha diversity

At 15 °C and 20 °C, the microbiome of *B. thuringiensis* Bt43-challenged larvae had a higher species abundance (8,606 and 18398.5, respectively) than *M. anisopliae* ICIPE 7-challenged larvae (6,992 and 13356.17, respectively). At 25 °C, the microbiome of ICIPE 7-challenged larvae had a higher species abundance (21557.5) than that of the Bt43-challenged FAW larvae (8,808) (Figure 4).

The microbiome of FAW larvae challenged with both pathogens (ICIPE 7 and Bt43) showed higher species richness than the control groups on day 0 (2 h post-treatment) and day 10 post-treatment across all temperatures (Figure 5). At 15 °C and 20 °C, FAW larvae inoculated with ICIPE 7 exhibited a higher species richness (342 and 440, respectively) compared to their counterparts infected with Bt43 (334 and 418) (Figures 5A,B). At 25 °C, the microbiome of Bt43-challenged larvae showed greater species richness (305) compared to that of the larvae infected with ICIPE 7 (226) (Figure 5C).

**FIGURE 5:**
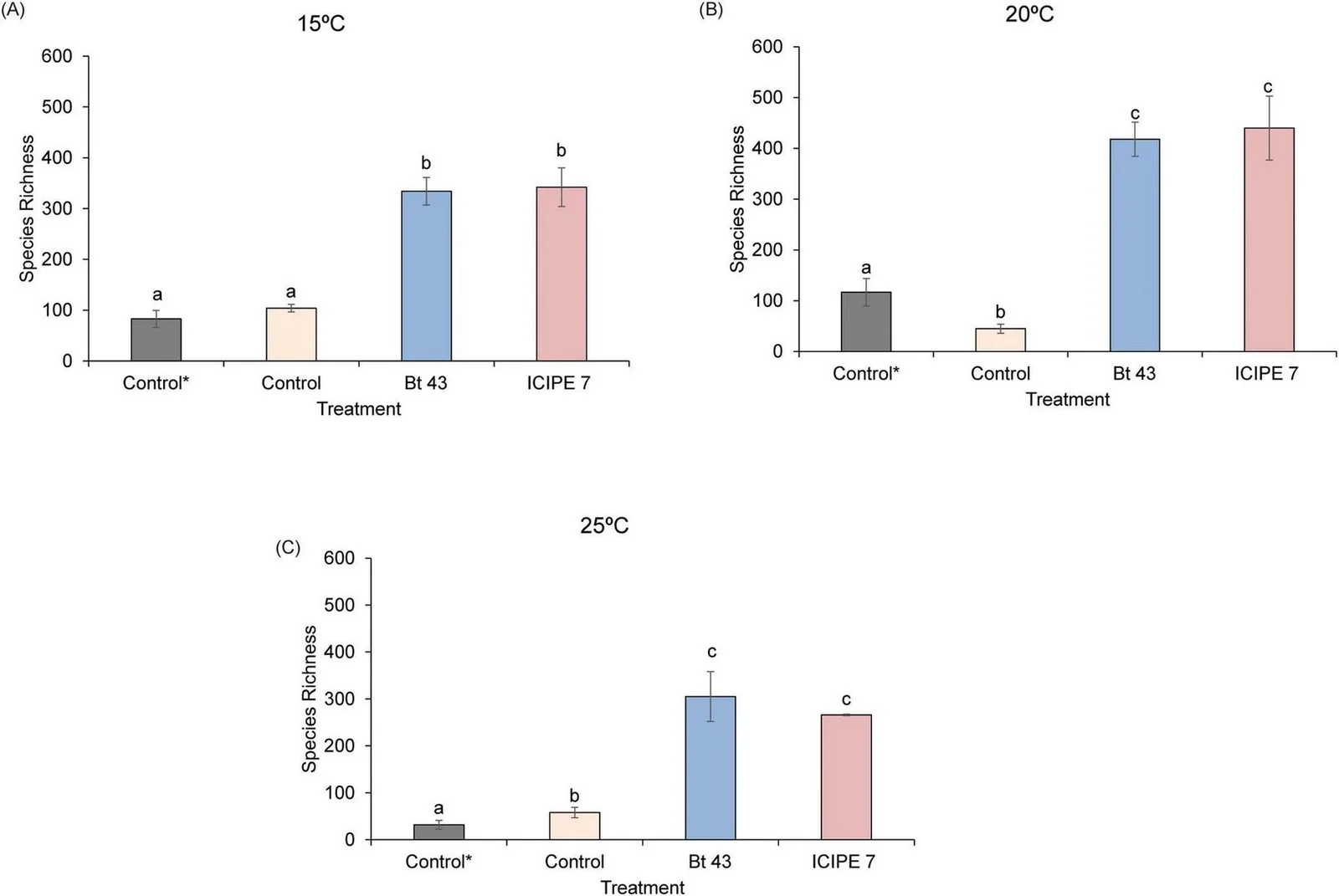
Microbiome richness at **(A)** 15 °C, **(B)** 20 °C, and **(C)** 25 °C of *Spodoptera frugiperda* larvae challenged with *Metarhizium anisopliae* isolate ICIPE 7, and *Bacillus thuringiensis* isolate Bt43, 10 days post-infection. Control*, control at day 0 in comparison to treatment at day 10 post exposure. The bars denote the means, and means followed by the same letters are not significantly different (at *P* < 0.05).

The Shannon-Weiner diversity metric accounts for gut microbiome richness and evenness. At 15 °C and 20 °C, the microbiome of FAW larvae challenged with ICIPE 7 showed a marginally greater Shannon diversity (3.359 and 2.837, respectively) compared to that of larvae challenged with Bt43 (2.837 and 2.87, respectively) (Figures 6A,B). At 25 °C, the Shannon diversity observed among larvae challenged with Bt43 was higher compared to that of larvae challenged with ICIPE 7 (2.691 and 2.487, respectively) (Figure 6C).

**FIGURE 6:**
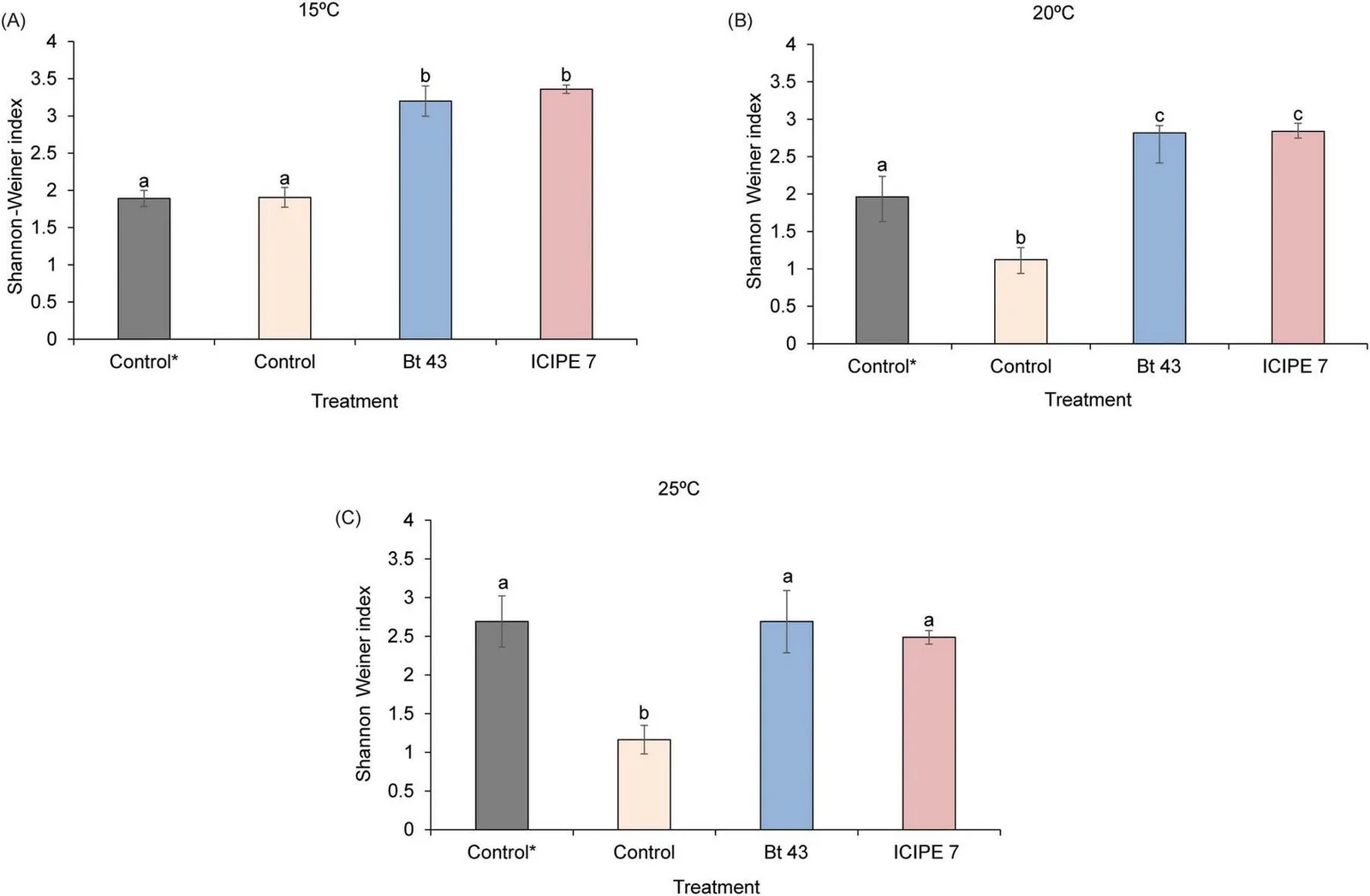
Alpha diversity at **(A)** 15 °C, **(B)** 20 °C, and **(C)** 25 °C of *Spodoptera frugiperda* larvae challenged with *Metarhizium anisopliae* isolate ICIPE 7, and *Bacillus thuringiensis* isolate Bt43, 10 days post-challenge, computed using the Shannon-Weiner Index. Control*, control at day 0 in comparison to treatment at day 10 post exposure. The bars denote the means, and means followed by the same letters are not significantly different (at *P* < 0.05).

#### 3.3.2 Beta diversity

The microbiome composition between the various treatments varied with changes in temperature and treatment, where FAW larvae challenged with both ICIPE 7 and Bt43 formed three clusters corresponding to 15 °C, 20 °C, and 25 °C (Figure 7).

**FIGURE 7:**
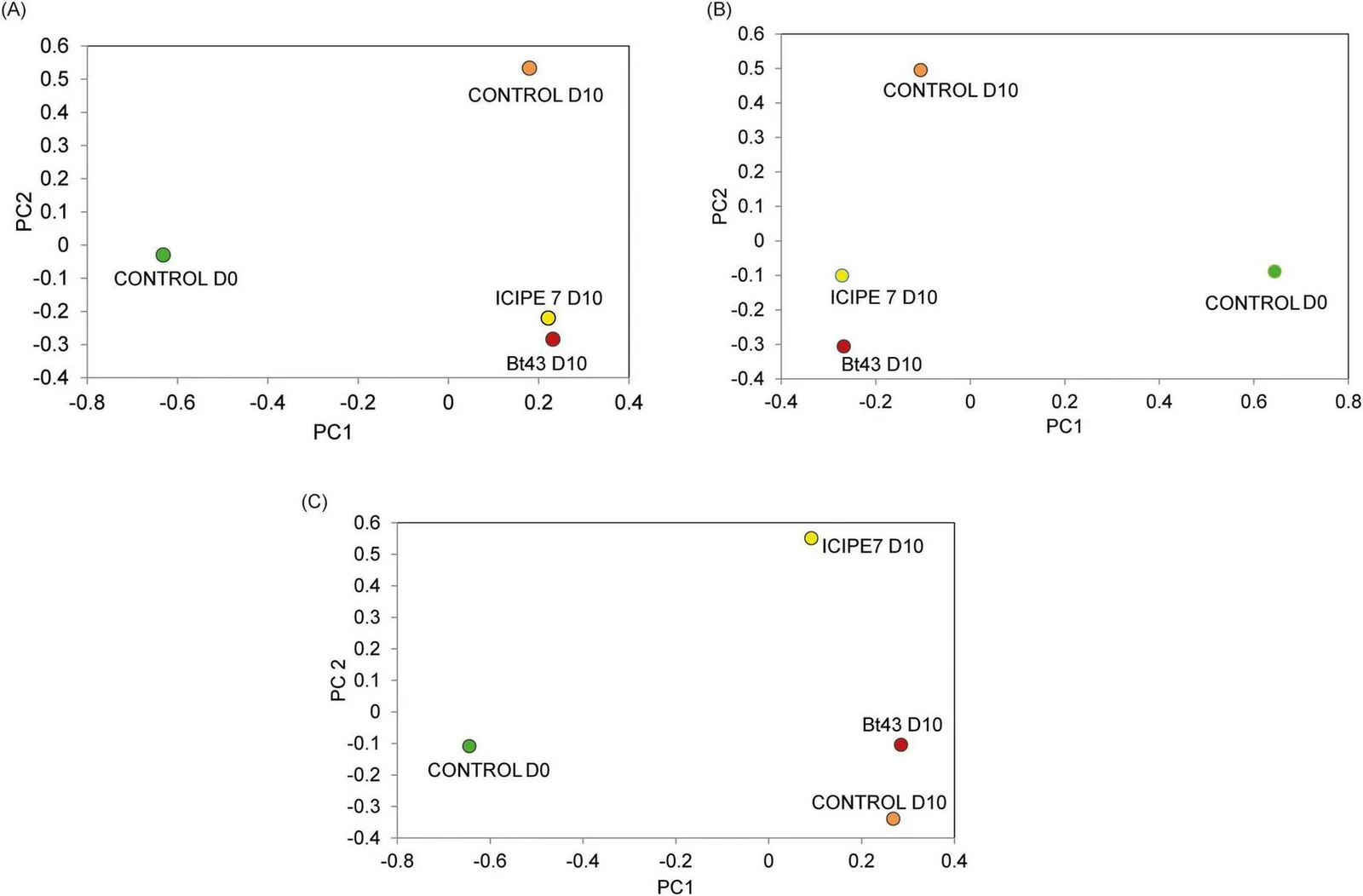
Two-dimensional principal coordinate plot of beta diversity among *Spodoptera frugiperda* larvae challenged with *Metarhizium anisopliae* isolate ICIPE 7 and *Bacillus thuringiensis* isolate Bt43 at 15 °C **(A)**, 20 °C **(B)**, and 25 °C **(C)**.

At 15 °C, the largest species diversity was noted between control larvae analyzed at day 0 and those examined on day 10 (98.74%) while the least was observed between ICIPE 7-challenged and Bt43-infected larvae at day 10 post-exposure (74.74%) (Figure 7A and Supplementary Table 2a). At 20 °C, the highest microbial diversity was observed between control larvae picked on day 10 and those challenged with Bt43 at day 10 post-infection (97.16%), while the least diversity was observed between ICIPE 7 and Bt43-infected larvae at 10 days post exposure (58.44%) (Figure 7B and Supplementary Table 2b). Finally, at 25 °C, the highest diversity was recorded between control larval groups assessed on day 0 and control larvae investigated on day 10 (99.89%), while the lowest was observed between control larvae picked on day 10 and those challenged with Bt43 on day 10 post-challenge (73.01%) (Figure 7C and Supplementary Table 2c).

## 4 Discussion

This study evaluated mortality, immune response (total and differential hemocyte counts), and changes in the gut microbiome of the economically important and invasive pest *S. frugiperda* upon infection with *M. anisopliae* and *B. thuringiensis*, two potential biopesticides to tackle the pest. The mortality data highlighted the significance of temperature as an important factor in determining the effectiveness or efficacy of biopesticides.

The mortality of FAW larvae infected with *M. anisopliae* isolates ICIPE 7 and ICIPE 78 demonstrated that these isolates are effective in managing this invasive pest. Our results are consistent with other studies that have shown *M. anisopliae* to be effective against FAW eggs and larvae under both laboratory and field conditions (Akutse et al., 2019; Cruz-Avalos et al., 2019; Lezama-Gutiérrez et al., 1996; Ramanujam et al., 2020; Romero-Arenas et al., 2014). Moreover, other studies have also demonstrated *M. anisopliae* to be effective against the larvae of invasive pests like *Chilo suppresalis* (striped rice stem borer) (Zibaee and Malagoli, 2014) and *Plutella xylostella* (diamond back moth) (Zafar et al., 2020).

Our findings have demonstrated that the efficacy of *M. anisopliae* isolates ICIPE 7 and ICIPE 78 is influenced by temperature. *M. anisopliae* host infection starts by having its spores attach themselves to the insect’s waxy epicuticle through hydrophobic and electrostatic forced coupled with protein interactions (Aw and Hue, 2017). With the carbon and nitrogen supply on the insect’s epicuticle, the spore is able to germinate and form a germ tube (Zibaee et al., 2013). After germination, the ODC1 and MpI1 genes specific to *M. anisopliae* facilitate the differentiation of the germ tube into an appresorium which uses mechanical pressure and enzymes such as chymotrypsins, carboxypeptidases, and trypsins to penetrate the insect’s cuticle (Aw and Hue, 2017; Seib et al., 2023). Upon penetration into the hemocoel, *M. anisopliae* starts to colonize its host’s hemolymph, with its hyphae multiplying and differentiating, until they extrude out through the cuticle into the environment (Aw and Hue, 2017). In 3–10 days, the host dies from water and nutrient loss, mechanical damage of organs, and toxin activity (Boaventura and Quintela, 2025).

These infection steps are mediated by enzymatic activities that are sensitive to factors such as pH, relative humidity, and temperature (Goettel et al., 1989). Moreover, measuring how EPF grow under certain conditions provides valuable insights into their potential pathogenicity (Dimbi et al., 2004; Seib et al., 2023; Slowik et al., 2023). The sporulation and survival of EPF is affected by temperature, and these have been found to be most optimal at temperatures ranging between 25 °C and 30 °C (Bugeme et al., 2008; Dimbi et al., 2004; Kuboka, 2013). Since germination rate is reduced at 20 °C, it takes longer for EPF spores to penetrate the host and thus delays the onset of disease (Dimbi et al., 2004). The rate of conidial growth was reported by Yeo et al. (2003) to be hampered at temperatures 10 °C and 15 °C; and since conidia are the infection units of EPF, reduced conidiation activity may result in reduced pathogenicity. The mortality of *S. frugiperda* observed during our study where mortality increased notably with increase in temperature may be explained by the increase in growth and sporulation of *M. anisopliae* and thus increase in pathogenicity with temperature dependence.

Studies against adult houseflies *Musca domestica*, with *M. anisopliae* also revealed similar outcomes to our work, where virulence was slower at 20 °C compared to 25 °C and 30 °C. The authors found that less than 25 spores were required to kill the insects at 25 °C, while they needed 5,896 spores to kill the adult fruitflies and over 100,000 spores to kill house flies at 20 °C in 9 days (Carswell et al., 1998). Maingi et al. (2023) also confirmed that *M. anisopliae* isolates ICIPE 18 and 20 had the highest virulence at 25 °C against *Phthorimea absoluta* adults. Higher percentage mortality and short lethal times were also observed when Tephritid fruit flies like the melon fly (*Zeugodacus cucurbitae* (Coquillett) (Diptera: Tephritidae) were treated with *M anisopliae* isolates ICIPE 18 and ICIPE 69 at 25 °C and 30 °C (Onsongo et al., 2019). Studies conducted with the *M anisopliae* isolate ICIPE 69 against adult western flower thrips [*Frankliniella occidentalis* Pergande (Thysanoptera: Thripidae)] recorded 100% mortality in 6 days at optimal temperature (Kuboka, 2013).

Despite FAW’s optimal temperature for survival and development being 28 °C (CABI, 2019) and with the larvae found to develop faster at 30 °C (Du Plessis et al., 2020; Huang et al., 2021), *M. anisopliae* isolates ICIPE 7 and ICIPE 78 were still highly virulent at a temperature range between 25 °C and 30 °C, making them a reliable option to be developed as a biopesticide against FAW. However, since the effectiveness of EPF is dependent on temperature, the prevailing climate in a farming area should be considered when considering their use (Maingi et al., 2023).

The efficacy of *B. thuringiensis* against FAW had been considered questionable (Bohorova, 1996, and studies with different *B. thuringiensis* isolates yielded inconsistent results, with some isolates showing high virulence against FAW while others caused very low mortalities (Li et al., 2019; Valicente and Barreto, 2003). However, contrary to the findings of Polanczyk et al. (2000), who reported that second instar FAW mortalities ranged between 70% and 100%, our bioassays with *B. thuringiensis* isolates Bt43 and HD263 against the same instar did not cause high mortality rates. These low mortality rates observed in this study are more consistent with Valicente and Barreto (2003), who recorded FAW larvae mortalities between 0% and 20% from 2799 Bt isolates. Fall armyworm adaptability to its environment might have contributed to the buildup of resistance against some *B. thuringiensis* isolates. Such resistance has been observed in some areas with transgenic crops (Bt cotton and maize) in pests such as diamondback moth *Plutella xylostella* (Linnaeus) (Lepidoptera: Plutellidae), western corn rootworm, *Diabrotica virgifera virgifera* LeConte (Coleoptera: Chrysomelidae), and fall armyworm (Afzal et al., 2024; Burtet et al., 2017; Li et al., 2020), which may explain the low mortalities observed. It is important to highlight that the insecticidal activity of *B. thuringiensis* against FAW and other lepidopteran pests varies with the type of toxic crystalline protein inclusions (Afzal et al., 2024; Li et al., 2019; Liu et al., 2024). *Spodoptera frugiperda* populations have been reported to possess low susceptibility to Bt proteins Cry1Ab and Cry1Ac. However, field-evolved resistance cases have been found in Puerto Rico against Cry1F, Cry1Ac and Cry1Ab, in Brazil against Cry1F and Cry1Ab, in Southeastern United States against Cry1A.105 and Cry1F; and in Argentina against Cry1F (Gutierrez-Moreno et al., 2020). Thus, the low mortalities observed among FAW larvae-challenged with *B. thuringiensis* isolates Bt43 and HD263 may be explained by these Bt isolates either expressing low-virulence toxins or the FAW having developed resistance against them. Immune priming, where exposure to sublethal concentrations of a pathogen can confer a memory-like immune protection against the host that can be passed to subsequent generations has also been implicated in resistance to Bt in pests like *Tenebrio molitor* Linnaeus (Coleoptera: Tenebrionidae) and *Galleria mellonella* (Linnaeus) (Lepidoptera: Pralidae) (Xiao et al., 2023).

In addition to resistance, the effectiveness of Bt against lepidopteran pests like *S. frugiperda* is also affected by temperature because some Bt insecticidal proteins’ stability is affected by change in temperature (González-Rizo et al., 2019). Studies against 3^rd^ instar mosquito larvae (*Aedes aegypti*) demonstrated variations in pathogenicity of Bt at 20 °C, 25 °C, and 30 °C, where pathogenicity increased with increase in temperature (González-Rizo et al., 2019; Lysyk and Selinger, 2012). On the other hand, studies against stable fly (*Stomoxys calcitrans*) larvae showed a reduction in mortality with an increase in temperature which may have been caused by an improvement in larval immunity with increase in instar and impact of temperature on the Bt toxins, despite the increased rate of feeding and consequent increase of Bt toxins consumed (Lysyk and Selinger, 2012). In our study, behavioral changes, such as reduced feeding by FAW larvae at 15 °C and 20 °C, may also have contributed to the low mortality rates attributed to Bt isolates (as the larvae did not consume sufficient Bt-inoculated maize leaves at 15 °C and 20 °C). For Bt toxins to be expressed, the target organism (FAW) must ingest sufficient Bt cells, which, in turn, express the cry proteins in the alkaline gut environment, leading to toxin production that causes gut paralysis and eventually leads to the target organism’s death (Li et al., 2020; Ruiu, 2015). Our study observed that despite FAW larvae’s feeding habits improving at the higher temperatures (25 °C and 30 °C), there were still low mortalities attributed to the tested Bt isolates; hence we postulate that the FAW’s immune system may have countered *B. thuringiensis* infection as the pest thrives at 28 °C (CABI, 2019). Similar observations have also been reported by Zhao et al. (2025) that *Cordyceps militaris* fungi induce feeding and weight gain in silkworm larvae using an insect-like trehalase, and where the promotion of caterpillar feeding and weight gain benefits fungal fruiting.

The cellular immune response plays a crucial role in the pathogenicity of biopesticides and may affect their effectiveness and application procedures (Fenibo and Matambo, 2025; Li et al., 2020; Malinga et al., 2022; Sta̧czek et al., 2020). We observed a decline in the Total Hemocyte Count (THC) in FAW larvae challenged with *M. anisopliae* isolates from days 0 to 1 post-infection, which may be explained by the fungus’s release of cytotoxic metabolites (Destruxins A and E, Cyclosporine, and Beauverolide) upon infection (Cao et al., 2016; Chen et al., 2014; Fan et al., 2013; Kershaw et al., 1999). A reduction in THC may also be explained by nodulation, a major cellular immune response in insects against invading fungal spores (Gillespie et al., 2000; Khosravi et al., 2013). This drop in THC 24 h post-infection was also observed in third-instar FAW larvae challenged with *Metarhizium rileyi* (Wang J. L. et al., 2022). A subsequent rise in THC on days 5 and 10 may indicate that the larvae were able to overcome infection by *M. anisopliae*, explaining their survival up to these days (Sta̧czek et al., 2020). Increase in THC may also mean that more hemocytes were deployed to counter *M. anisopliae* invasion, as was observed in studies with the EPF *Tolypocladium cylindrosporum* against *G. mellonella* larvae; and *B. bassiana* and *Nomuraea rileyi* against *Spodoptera littoralis* (Boisduval) (Lepidoiptera: Noctuidae) larvae (Bandani, 2008; Meshrif et al., 2011). Hemocyte density may also have increased with a change in instar because THC increases with larval mass (Gardiner and Strand, 2000). Gardiner and Strand (2000) also reported that hemocyte counts depend on cell turnover rate, mitosis, cell release rate from hematopoietic organs, and stressors such as infection and wounding.

The THC trend for FAW larvae infected with *B. thuringiensis* was not as pronounced as that of those observed in larvae infected with *M. anisopliae*. Studies to track THC upon exposure to *B. thuringiensis* with various pest species also yielded varying results. For instance, in Egyptian cotton leafworm, *S. littoralis* larvae, exposure to *B. thuringiensis* increased THC levels (Vengateswari et al., 2020). However, in palm weevil, *Rhynchophorus ferrugineus* (Olivier) (Coleoptera: Curculionidae) larvae, there was a decrease in THC upon exposure to sub-lethal doses of *B. thuringiensis* (Celi et al., 2022). Furthermore, there was a decline in THC observed in greater wax moth, *G. mellonella* larvae 24 h post-exposure to *B. thuringiensis*, followed by a rise at 48 and 72 h post-exposure (Tunçsoy and ÖZALP, 2021). A decline in THC may also be explained by nodulation, which insects apply as a primary defense against bacterial infection as it allows for fast elimination of numerous bacterial spores from the hemolymph (Cho and Cho, 2019) while an increase in THC with time is an indication of successful suppression of invading pathogens by the host insects’ immune system (Sta̧czek et al., 2020); mechanism which was also observed in FAW larvae infected with both *M. anisopliae* and *B. thuringiensis* on days 5 and 10.

Granulocytes and plasmatocytes were described as making up more than 50% of the circulating hemocytes in lepidopteran larval stages (Dongxu et al., 2016) which is consistent with our differential hemocyte count results that showed that the hemocyte types in FAW are Plasmatocytes and Granulocytes. Our findings are also consistent with those from studies with other lepidopteran species such as *G. mellonella* (Senior and Titball, 2020; Sta̧czek et al., 2020; Wu et al., 2016) and *S. littoralis* (Zibaee et al., 2013). In another study to determine the immune response of striped rice stem borer, *Chilo suppressalis* (Walker) (Lepidoptera: Crambidae) larvae exposed to *M. anisopliae* also found that Prohemocytes, Plasmatocytes, Granulocytes, and Oenocytoids were the most abundant circulating hemocytes (Zibaee and Malagoli, 2014).

In our study, the numbers of hemocytes were low among FAW larvae treated with *M. anisopliae* ICIPE 7 and ICIPE 78, but the few hemocytes we observed were mostly plasmatocytes and granulocytes. These numbers fluctuated with time and treatment, where we recorded increases in plasmatocytes and drops in granulocytes and vice-versa, without any notable pattern, while Oenocytoids, Spherulocytes, and Adipohemocytes remained rare to find.

The massive numbers of granulocytes and plasmatocytes found in FAW hemolymph suggests that these hemocyte types play key roles in the pest’s cellular immune response. Insects defend themselves against infection by bacteria using cellular immune defenses that have been shown to slow or stop the progress of disease (Celi et al., 2022). Hemocytes may adhere to the surface of invading EPF hyphae and thus slow or stop the onset of disease (Maingi et al., 2023). Granulocytes and plasmatocytes are the only hemocytes that can adhere to foreign particles and facilitate their recognition and elimination (Dongxu et al., 2016). When granulocytes detect a foreign body, they lyse and degranulate, releasing compounds that trigger plasmatocytes to attach themselves onto the foreign body (Cho and Cho, 2019), an immune response that may reduce their numbers in the hemolymph. During infection with *M. anisopliae*, the fungus releases cytotoxic metabolites like Destruxins in an attempt to release encapsulated spores, and these are known to reduce the number of plasmatocytes and granulocytes (Fan et al., 2013; Lavine and Strand, 2002). A reduction in plasmatocytes may indicate an increase in nodulation against *M. anisopliae* and Bt infection because they are the key component in nodule formation (Sta̧czek et al., 2020) or an increase in selective plasmatocyte apoptosis (Maingi et al., 2023). Granulocytes also take part in nodulation, which reduces their numbers in hemolymph when they form aggregates around invaders such as fungal spores or entrap bacteria in mucopolysaccharide matrices (Celi et al., 2022; Suwannakhon et al., 2024). The subsequent increase in plasmatocytes and granulocytes at days 5 and 10 post-infection may be explained by an increase in larval immunity with time and instar, whereby mobilized hematopoietic tissues released more of these cells to aid in wound healing and other immune defenses, thus enabling the larvae examined to survive to these days post-infection (Eleftherianos et al., 2021; Lysyk and Selinger, 2012).

Studies against mosquitos (*Aedes aegypti*) with *B. bassiana* showed an increase in granulocytes and plasmatocytes, while against housefly larvae (*Musca domestica*), the number of granulocytes and oenocytoids dropped 24 h post-infection (de Oliveira Barbosa Bitencourt et al., 2023). Fiorotti et al. (2019) also observed an increase in the number of granulocytes and plasmatocytes in ticks (*Rhipicephalus microplus*) 24 h post-treatment with *M robertsii*. Suwannakhon et al. (2024) observed different DHCs in Mealworm beetle (*Tenebrio molitor*) larvae treated with *B. bassiana* and Bt, where the number of granulocytes increased 24 and 72 hpi with Bt but and was observed to drop 120 and 168 hpi with *B. bassiana*; while the number of plasmatocytes remained high throughout after infection with both Bt and *B. bassiana*.

We also found prohemocytes in *S. frugiperda* larvae in low numbers compared to plasmatocytes and granulocytes. While their role in *S frugiperda* is not known, these have a high mitotic index and are regarded as the stem cells of all hemocytes (Brehelin and Zachary, 1986). We also spotted few oenocytoids, which are believed to play a role in wound healing and phenoloxidase activity, key immune responses against invasion by EPF conidia (Brehelin and Zachary, 1986; Moyetta et al., 2021). Spherulocytes were also present in small numbers, and their role in immunity is also mostly unknown. A study with *Bombyx mori* suggests that they may play a role in silk production as there was a correlation between their numbers and the quantity of silk the larvae produced (Sta̧czek et al., 2020). Their spherule inclusions, which are released when they lyse, may also be involved in the transport of cuticle elements (Brehelin and Zachary, 1986). Adipohemocytes, which were also rare, have massive lipid droplets in their cytoplasm which when combined with RER and Golgi body cisternae are indicative of secondary cellular functions. Other studies suggest that they may be fat body cells released into circulation at the time of hemolymph extraction, which may explain their appearance (Lawrence, 2008).

The FAW larval gut microbial composition may have played an important role in the pest’s susceptibility or resistance to biopesticides. We observed that Firmicutes (Bacillota) and Proteobacteria were the most abundant phyla in the FAW larvae infected with either *B. thuringiensis* or *M. anisopliae* isolates and control larvae examined on day 0 and day 10. Under Firmicutes, we found the orders Eubacteriales, Lactobacillales, and Bacillales, while the most abundant orders in the phylum Proteobacteria were Enterobacteriales and Pseudomonadales. These findings are consistent with the reports on FAW larvae and adults by Gichuhi et al. (2020), Lv et al. (2021), Yuning et al. (2022). These bacterial species were also found in Lepidoptera, including *Spodoptera exigua* (Hübner) (Lepidoptera: Noctuidae) (Chen et al., 2022; Gao et al., 2019) and *Lymantria dispar* (Linnaeus) (Lepidoptera: Erebidae) (Zeng et al., 2020). At the genus level, *Klebsiella*, *Serratia*, *Enterococcus*, *Wolbachia*, and *Escherichia* were the most abundant in both inoculated and control larvae.

While the clear beneficial roles of bacteria in these orders have not been determined for fall armyworm (Mason et al., 2021), they have been implicated in defense against pathogen infection in other insects such as *B. mori* (Linnaeus) (Lepidoptera: Bombycidae) (Zeng et al., 2022), *L. dispar*, and *S. littoralis* (Voirol et al., 2018) and nutrition, including cellulose digestion in *B. mori* (Zhang et al., 2022), nitrogen fixation in *P. xylostella* (Voirol et al., 2018) and vitamin synthesis in *Glossina brevipalpis* Newstead (Diptera: Glossinidae) (Krishnan et al., 2014). Given these functions’ critical roles in FAW survival, we postulate that the abundance of these bacteria orders in the FAW microbiome may be crucial for their survival against pathogen infection.

We observed elevation in the evenness of FAW microbiome 10 days post-infection with *M. anisopliae* and Bt43, which suggests that infection with ICIPE 7 at 15 °C and 20 °C and infection at 25 °C with Bt caused different species in FAW microbiome to adapt in equal abundance. A low evenness index suggests that a microbiome composition is more heterogeneous (Ajene et al., 2020; Magurran, 2013). The microbiome of FAW larvae infected with ICIPE 7 at 15 °C and 20 °C and those infected with Bt43 at 25 °C 10 days post-infection, had a richer bacterial community compared to their counterparts infected with the opposite treatment, which may indicate that infection with these entomopathogens caused the microbiome to become more complex. We also postulate that with time and as temperature increases, the infected FAW larvae microbiome becomes more diverse and complex. The highest intercommunity diversity observed between the treatments and control group samples (100% diversity) indicates that infection by *M. anisopliae* isolate ICIPE 7 and *Bacillus thuringiensis* isolate Bt43 did disrupt FAW microbiome diversity. It was reported that *M. anisopliae* infection also caused a shift in the bacterial community structure of brown planthopper, *N. lugens* nymphs compared to controls (Wang Z. et al., 2022). The findings of our study showed that alpha and beta diversity are similar to those observed in *S. exigua* larvae, where the alpha-diversity indices Richness, Evenness, Shannon diversity, and Simpson diversity did not vary among infected larvae at 10 °C, 26 °C (control group), and 35 °C, but notable variations were observed in beta-diversity, suggesting that temperature may affect microbiome structure in insects (Chen et al., 2022). Some factors that can cause intercommunity diversity include resistance to insecticides, changes in instar, diet, exposure to pesticides, and habitat (Indiragandhi et al., 2007; Voirol et al., 2018). Infection by EPF such as *M. anisopliae* and *B. bassiana* causes changes in bacterial load, microbiome composition and diversity, as well as activity of various species as reported in brown planthopper *N. lugens* nymphs and female adult mosquitoes *A. stephensi* Liston (Diptera: Culicidae) (Wang Z. et al., 2022; Wei et al., 2017). In Gypsy moth *L. dispar* larvae, indigenous gut bacteria are required for the toxic activity of *B. thuringiensis* (Broderick et al., 2009). However, treatment with *B. thuringiensis* was also found to cause a notable disruption of the larval gut microbiota of Lepidoptera species such as *S. exigua* and *S. littoralis* (Caccia et al., 2016; Li et al., 2022). In these studies, a reduction in microbiome diversity was associated with the inability to survive infection by EPF and *B. thuringiensis*. However, in this current study, microbiome diversity was greater in infected larvae than in controls, suggesting that bacterial species may have played a role in ensuring their survival up to day 10 post-infection. Bacterial species such as *Pseudomonas* interfere with the growth and differentiation of fungi, which suggests they aid in immunity against EPF in insects such as Colorado potato beetle *Leptinotarsa decemlineata* (Say) (Coleoptera: Chrysomelidae), onion fly *Delia antiqua* (Meigen) (Diptera: Anthomyiidae), and leaf-cutting ant *Atta cephalotes* (Linnaeus) (Hymenoptera: Formicidae) (Boucias et al., 2018; Kryukov et al., 2021). However, further studies on lepidopteran insects with *M. anisopliae* and *B. thuringiensis* may provide additional information or insights on their effects on microbiome communities.

## 5 Conclusion

The findings of this study demonstrate that *M. anisopliae* isolates ICIPE 7 and ICIPE 78 are strong candidates for use in FAW integrated pest management and that temperature has notable consequences on the effectiveness of these microbial biopesticides against FAW. In addition, to effectively incorporate *B. thuringiensis* isolates into FAW-IPM, additional studies are needed to select isolates with potent Cry and Vip protoxins that remain effective against FAW as well as assessing dose responses of these potent isolates at the optimal temperature. The study has also demonstrated that Plasmatocytes and Granulocytes, which play key roles in nodulation, encapsulation, and phagocytosis, are the most abundant hemocytes in FAW larvae. Moreover, we demonstrate that EPF and *B. thuringiensis* infection increased bacterial load and intercommunity diversity in the FAW larvae microbiome.

## Data Availability

The data presented in the study are deposited in GenBank, NCBI, under BioProject accession number PRJNA1518384.

## Footnotes

1
https://epi2me.nanoporetech.com/
