# Supplementary material for: Immunological responses and gut microbial shift of fall armyworm Spodoptera frugiperda challenged with entomopathogens
Source: Front Microbiol. 2026 Sep 14;17:1929843. doi: 10.3389/fmicb.2026.1929843 (PMC13617015; doi:10.3389/fmicb.2026.1929843)
Supplement: Supplementary file 1 [file Table_1.docx]

**Immunological responses and gut microbial shift of fall armyworm (*Spodoptera frugiperda*) challenged with entomopathogens under changing temperatures**

Martha W. Nderitu^1,2^, Fathiya M. Khamis^1,3*^, Inusa J. Ajene^1^, Steven G. Nyanjom^2^, Komivi S. Akutse^1,4*^

^1^International Centre of Insect Physiology and Ecology (*icipe*), Nairobi, Kenya.

^2^Jomo Kenyatta University of Agriculture and Technology (JKUAT), Nairobi, Kenya.

^3^Department of Zoology and Entomology, University of Pretoria, Hatfield, South Africa.

^4^Unit for Environmental Sciences and Management, North-West University, Potchefstroom, 2520, South Africa.

^*^Corresponding author: [fkhamis@icipe.org](mailto:fkhamis@icipe.org); [kakutse@icipe.org](mailto:kakutse@icipe.org)

**Figure S1:** Plasmatocytes from *Spodoptera frugiperda* larvae

**Figure S2:** Prohemocytes from *Spodoptera frugiperda* larvae.

**Figure S3:** Granulocytes from *Spodoptera frugiperda* larvae.

**Figure S4:** Spherulocytes from *Spodoptera frugiperda* larvae.

**Figure S5:** Oenocytoids from *Spodoptera frugiperda* larvae

**Figure S6:** Adipohemocytes from *Spodoptera frugiperda* larvae

**Figure S7:** Phenoloxidase activity observed as optical density (OD) average at 490 nm in the hemolymph of *S. frugiperda* larvae infected with *Metarhizium anisopliae* isolate ICIPE 7 at 25 °C evaluated over 30 minutes on days 0 (A), 1 (B), 5 (C), and 10 (D) and average PO activity observed as optical density (OD) average at 490 nm in the hemolymph of *S. frugiperda* larvae infected with *Metarhizium anisopliae* isolate ICIPE 7 at 25 °C at days 0, 1, 5, and 10 (E).

**Figure S8:** Cumulative abundance of bacterial species in FAW larvae infected with *Metarhizium anisopliae* isolate ICIPE 7 and *B. thuringiensis* isolate Bt43 10 days post exposure at different temperature regimes (15 °C, 20°C, and 25°C).

**Table S1a:** Table showing the relative percentages of hemocytes in *Spodoptera frugiperda* larvae infected with *Metarhizium anisopliae* strains ICIPE 78, ICIPE 7 and *B. thuringiensis* strains Bt43 and HD263, and Control treatments at 15 °C. For each hemocyte type, percentage means with different lowercase letters in each column across infected/control groups within the same day and different uppercase letters in each row across different days for each treatment/control groups denote a significant difference (p ≤ 0.05, n=90)

|  | **Day (D)** | **Prohemocytes** | **Plasmatocytes** | **Granulocytes** | **Spherulocytes** | **Oenocytoids** | **Adipohemocytes** |
| --- | --- | --- | --- | --- | --- | --- | --- |
| ICIPE 78 | D0 | 3.162±0.45Aa | 36.03±3.97Ab | 60.34±4.44Ba | 0.233±0.01Aa | 0.233±0.01Aa | 0.00Aa |
|  | D1 | 8.082±0.69Cb | 34.70±4.43Bb | 56.78±3.74Ca | 0.218±0.001Aa | 0.2183±0.001Aa | 0.00Aa |
|  | D5 | 5.347±0.96Bb | 14.92±0.31Aa | 79.38±0.59Dc | 0.00Aa | 0.349±0.06Bb | 0.00Aa |
|  | D10 | 3.089±0.39Aa | 46.68±0.6Cc | 46.49±0.87Bb | 0.1467±0.004Aa | 0.658±0.05Bc | 2.929±0.06Ab |
| ICIPE 7 | D0 | 8.152±0.94ABb | 30.24±0.11Cc | 60.72±0.98Ab | 0.00Aa | 0.893±0.15Cb | 0.00Aa |
|  | D1 | 3.954±0.63Aa | 4.876±0.86Aa | 90.43±0.4Cc | 0.00Aa | 0.740±0.17Bab | 0.00Aa |
|  | D5 | 17.08±3.21Bb | 75.40±1.49Cc | 6.738±1.67Aa | 0.00Aa | 0.7825±0.06Ba | 0.00Aa |
|  | D10 | 8.501±0.91Ab | 10.99±4.45Ab | 76.33±3.42Bc | 0.00Aa | 0.813±0.2Bb | 3.359±0.32Cb |
| Bt43 | D0 | 4.141±0.76ABa | 27.52±1.61Bb | 68.07±2.39Cc | 0.00Aa | 0.274±0.02Ba | 0.00Aa |
|  | D1 | 6.693±1.60BCa | 25.52±1.69Ba | 67.28±0.04Cb | 0.00Aa | 0.515±0.12Bab | 0.00Aa |
|  | D5 | 7.065±1.33Ba | 24.31±2.44ABa | 68.21±1.12Bb | 0.2038±0.01Aa | 0.2038±0.01Ab | 0.00Aa |
|  | D10 | 18.89±0.91Bb | 32.61±5.56Ba | 44.87±5.13Aa | 0.715±0.23Bb | 0.557±0.07Ac | 2.349±0.17Cb |
| HD263 | D0 | 5.668±0.28BCc | 28.58±0.51Db | 65.66±0.79Dc | 0.00Aa | 0.094±0.00004Aa | 0.00Aa |
|  | D1 | 3.397±0.44BCb | 28.90±2.86Cb | 67.41±2.35Db | 0.113±0.0034Aa | 0.171±0.06ABab | 0.00Aa |
|  | D5 | 2.861±0.40Aa | 26.18±2.81Ba | 70.33±2.32Ca | 0.178±0.003Aa | 0.4475±0.1Bb | 0.00Aa |
|  | D10 | 2.625±0.32Ab | 39.82±0.07Dc | 54.98±0.52Cb | 0.327±0.04Cb | 0.422±0.05Bc | 1.829±0.26BCb |
| Control | D0 | 9.113±2.3Cb | 26.06±0.37Bc | 64.31±1.9Cc | 0.1302±0.01Aa | 0.3905±0.02Ca | 0.00Aa |
|  | D1 | 8.413±2.7ABa | 51.11±0.32Bb | 40.48±2.38Aa | 0.00Aa | 0.00Aa | 0.00Aa |
|  | D5 | 3.180±0.77Aa | 20.22±0.26Aa | 76.40±0.51BCb | 0.2044±0.0035Aa | 0.00Aa | 0.00Aa |
|  | D10 | 9.701±1.9Aa | 50.62±0.98Bb | 36.48±2.88Aa | 0.00Aa | 0.755±0.05Ab | 2.441±0.04ABb |

**Table S1b:** Table showing the relative percentages of hemocytes in *Spodoptera frugiperda* larvae infected with *Metarhizium anisopliae* strains ICIPE 78, ICIPE 7 and *B. thuringiensis* strains Bt43 and HD263, and Control treatments at 20 °C. For each hemocyte type, percentage means with different lowercase letters in each column across infected/control groups within the same day and different uppercase letters in each row across different days for each treatment/control groups denote a significant difference (p ≤0.05, n=90)

|  | **Day (D)** | **Prohemocytes** | **Plasmatocytes** | **Granulocytes** | **Spherulocytes** | **Oenocytoids** | **Adipohemocytes** |
| --- | --- | --- | --- | --- | --- | --- | --- |
| ICIPE 78 | D0 | 8.693±0.56Ab | 43.34±0.28Ab | 47.63±0.93Aa | 0.00Aa | 0.34±0.10ABa | 0.00Aa |
|  | D1 | 10.71±1.05Aa | 27.47±3.21Aa | 57.99±2.31Ca | 2.239±0.03Cb | 0.936±0.20ABa | 0.65±0.08Aa |
|  | D5 | 5.187±1.24ABb | 56.02±0.4Bc | 37.84±1.41Aa | 0.00Aa | 0.95±0.23Cb | 0.00Aa |
|  | D10 | 5.320±0.6Ab | 54.18±5.82BCc | 37.52±7.11Aa | 0.00Aa | 0.00Aa | 2.981±0.69Ca |
| ICIPE 7 | D0 | 6.286±1.07Aa | 38.96±3.46Ab | 53.88±4.75ABab | 0.1897±0.03Aa | 0.677±0.19Ca | 0.00Aa |
|  | D1 | 12.48±2.37Ba | 5.9340±0.32Aa | 80.06±2.71Ba | 0.00Aa | 0.9605±0.21Ca | 0.570±0.18Aab |
|  | D5 | 7.254±0.73BCa | 36.81±1.12Ab | 54.67±1.85Bb | 0.1813±0.0002Aa | 0.6347±0.09ABCa | 0.453±0.09Bbc |
|  | D10 | 17.61±1.08Bb | 21.34±1.69Aa | 59.19±0.73ABCb | 0.00Aa | 1.1707±0.21BCb | 0.681±0.09Ac |
| Bt43 | D0 | 6.099±1.98Ab | 15.96±1.21Aa | 77.80±3.34Cc | 0.00Aa | 0.144±0.14ABb | 0.00Aa |
|  | D1 | 24.85±3.49Cb | 59.33±0.11Aa | 15.82±3.6Aa | 0.00Aa | 0.00Aa | 0.00Aa |
|  | D5 | 3.846±0.07Aa | 24.46±3.17Aa | 70.77±3.35Bb | 0.00Aa | 0.4440±0.17Ac | 0.476±0.06Bb |
|  | D10 | 5.963±1.04Aab | 19.49±2.83ABa | 72.64±3.49BCb | 0.00Aa | 0.787±0.35Ba | 1.115±0.02Bc |
| HD263 | D0 | 8.824±0.59Aa | 19.35±0.74Ab | 71.34±1.39Cab | 0.00Aa | 0.141±0.001Bb | 0.353±0.07Aa |
|  | D1 | 3.028±0.26Aa | 29.88±0.96Bb | 66.62±1.4Dab | 0.00Aa | 0.272±0.02BCb | 0.199±0.05Aa |
|  | D5 | 5.386±1.04BCb | 22.40±2.40Aab | 71.50±1.35Cb | 0.1317±0.01Aa | 0.5857±0.002c | 0.00Aa |
|  | D10 | 4.018±1.08Aa | 23.65±4.24ABa | 70.61±2.91Ca | 0.00Aa | 0.00Aa | 1.721±0.25Cb |
| Control | D0 | 8.611±0.93Aa | 30.64±5.61Aa | 60.59±4.7BCab | 0.158±0.02Aa | 0.00Aa | 0.00Aa |
|  | D1 | 4.950±0.64BCa | 36.70±0.02Cb | 57.87±0.69Db | 0.2376±0.02Bb | 0.236±0.02BCab | 0.00Aa |
|  | D5 | 6.780±1.03Ca | 45.40±0.63Bb | 44.62±0.73Ba | 0.500±0.09Bc | 0.353±0.06ABb | 2.353±0.3Cb |
|  | D10 | 12.24±0.52Bb | 53.09±3.56Cc | 32.07±4.18ABa | 0.2818±0.04Bb | 0.791±0.07Cc | 1.534±0.02Cb |

**Table S1c:** Table showing the relative percentages of hemocytes in *Spodoptera frugiperda* larvae infected with *Metarhizium anisopliae* strains ICIPE 78, ICIPE 7 and *B. thuringiensis* strains Bt43 and HD263, and Control treatments at 25°C. For each hemocyte type, percentage means with different lowercase letters within each column across infected/control groups within the same day and different uppercase letters within each row across different days for each treatment/control groups denote a significant difference (p ≤0.05, n=90)

|  | **Day (D)** | **Prohemocytes** | **Plasmatocytes** | **Granulocytes** | **Spherulocytes** | **Oenocytoids** | **Adipohemocytes** |
| --- | --- | --- | --- | --- | --- | --- | --- |
| ICIPE 78 | D0 | 8.172±1.65Ba | 28.02±1.69Aa | 63.58±0.19Cb | 0.00Aa | 0.223±0.001ABa | 0.00Aa |
|  | D1 | 5.281±1.35Aa | 32.88±0.29Aa | 59.47±1.46Aa | 0.261±0.02Aa | 2.103±0.38Cc | 0.00Aa |
|  | D5 | 2.759±1.21Aa | 22.96±1.23Aa | 73.6±0.12Cc | 0.138±0.01Aa | 0.00Aa | 0.544±0.1Db |
|  | D10 | 2.32±1.21Aa | 15.02±1.55Aa | 81.57±2.75Dd | 0.00Aa | 0.447±0.06Cb | 0.645±0.04Bc |
| ICIPE 7 | D0 | 6.9±1.55Bb | 20.36±1.84Aa | 72.56±0.28Bb | 0.00Aa | 0.169±0.01Aa | 0.00Aa |
|  | D1 | 11.11±0.06Bc | 45.09±1.07Cb | 43.81±1.13ABa | 0.00Aa | 0.00Aa | 0.00Aa |
|  | D5 | 1.62±0.10Aa | 35.93±2.32Bb | 61.58±2.47Bb | 0.135±0.0029Aa | 0.27±0.01Bb | 0.47±0.06CDc |
|  | D10 | 3.365±0.13Ab | 45.31±0.17Bc | 50.84±0.33Cb | 0.00Aa | 0.266±0.04Bb | 0.214±0.01Ab |
| Bt43 | D0 | 5.305±0.74Bb | 23.44±1.56Aa | 70.82±0.71BCc | 0.00Aa | 0.436±0.1BCab | 0.00Aa |
|  | D1 | 4.085±0.43Aab | 49.69±1.46Cc | 45.97±0.97ABb | 0.00Aa | 0.257±0.07ABa | 0.00Aa |
|  | D5 | 8.031±0.34Aa | 17.2±1.30Aa | 70.99±1.83Aa | 1.174±0.15Bb | 1.43±0.11Cb | 1.174±0.15BCb |
|  | D10 | 5.335±0.80Ab | 36.03±0.77Ab | 56.78±1.57Bb | 0.00Aa | 0.691±0.13BCb | 1.166±0.14Bc |
| HD263 | D0 | 5.764±0.53Bc | 32.89±2.28Bb | 60.77±1.61Cc | 0.144±0.0011Aa | 0.431±0.14Cab | 0.00Aa |
|  | D1 | 3.157±0.53Ab | 24.25±0.48Bb | 72.11±1.01Cd | 0.179±0.06Aa | 0.298±0.06Ba | 0.00Aa |
|  | D5 | 3.988±0.63Aa | 28.52±4.67Aa | 64.48±3.98Ab | 0.402±0.02Aa | 1.802±0.12Db | 0.805±0.04ABb |
|  | D10 | 20.89±1.28Bd | 27.38±2.04Aa | 46.61±0.45Aa | 0.00Aa | 1.785±0.48Cb | 3.343±0.73Cc |
| Control | D0 | 4.513±1.40Aa | 20.59±1.58ABa | 74.9±2.98Ac | 0.00Aa | 0.00Aa | 0.00Aa |
|  | D1 | 2.647±0.48Aa | 49.74±1.83Dc | 47.47±2.31Ba | 0.147±0.002Aa | 0.00Aa | 0.00Aa |
|  | D5 | 4.759±0.85Ba | 42.56±0.55Cb | 52.46±0.31Bb | 0.114±0.01Aa | 0.00Aa | 0.114±0.01Aa |
|  | D10 | 11.00±0.61ABb | 47.15±1.93Ac | 40.35±2.35Aa | 0.00Aa | 0.00Aa | 1.502±0.18Bb |

**Table S1d:** Relative percentages of hemocytes in *Spodoptera frugiperda* larvae infected with *Metarhizium anisopliae* strains ICIPE 78, ICIPE 7 and *B. thuringiensis* strains Bt43 and HD263, and Control treatments at 30 °C. For each hemocyte type, percentage means with different lowercase letters within each column across infected/control groups within the same day and different uppercase letters within each row across different days for each treatment/control groups denote a significant difference (p ≤0.05, n=90)

|  | **Days (D)** | **Prohemocytes** | **Plasmatocytes** | **Granulocytes** | **Spherulocytes** | **Oenocytoids** | **Adipohemocytes** |
| --- | --- | --- | --- | --- | --- | --- | --- |
| ICIPE 78 | D0 | 2.574±0.48Ba | 36.59±3.84Cb | 60.19±4.24Aa | 0.3125±0.04Bb | 0.187±0.05Aa | 0.1253±0.01Aa |
|  | D1 | 5.779±1.7Aab | 26.18±2.02Ba | 67.03±3.86Ca | 0.1447±0.0008Aa | 0.7228±0.14BCb | 0.1447±0.001Aa |
|  | D5 | 3.65±0.72BCb | 40.04±2.0Cc | 55.68±1.28Cb | 0.155±0.04Bb | 0.21±0.07Cab | 0.24±0.04Aab |
| ICIPE 7 | D0 | 4.873±0.73Ca | 19.63±4.09Aa | 74.69±4.72Bc | 0.00Aa | 0.124±0.01Aa | 0.6867±0.09Bb |
|  | D1 | 6.296±0.46Aa | 18.36±2.09ABa | 74.37±2.8Bb | 0.18±0.0029Aa | 0.630±0.1ABb | 0.1825±0.18ABa |
|  | D5 | 10.72±0.64Cb | 55.73±3.49Bb | 31.22±4.41Aa | 0.00Aa | 0.00Aa | 2.33±0.28Cc |
| Bt43 | D0 | 3.651±0.57Ba | 27.71±0.92ABa | 68.27±0.24Ac | 0.00Aa | 0.3719±0.12Ba | 0.00Aa |
|  | D1 | 7.086±0.21Ab | 17.24±1.12Cb | 69.93±0.68Ab | 0.00Aa | 0.4582±0.02Ac | 5.285±0.639Db |
|  | D5 | 7.716±0.18ABc | 52.67±1.38Bb | 35.66±1.65Aa | 0.193±0.01a | 0.2943±0.11ABb | 3.467±0.03BCb |
| HD263 | D0 | 1.626±0.12Aa | 34.22±0.48BCa | 64.16±0.6Ac | 0.00Aa | 0.00Aa | 0Aa |
|  | D1 | 4.804±0.30Ac | 46.24±0.74Db | 47.68±1.05Bb | 0.1157±0.0003Aa | 0.6367±0.06Cc | 0.521±0.06Cb |
|  | D5 | 2.882±0.21Ab | 61.76±1.12Cc | 34.00±0.78Ba | 0.00Aa | 0.2259±0.0029ABCb | 1.131±0.13Cc |
| Control | D0 | 6.090±0.43Db | 26.29±0.89BCc | 67.51±0.47Bc | 0.00Aa | 0.0562±0.06Aa | 0.057±0.06Aa |
|  | D1 | 4.918±0.08Aa | 14.56±0.97Aa | 78.90±0.98Db | 0.1406±0.002Aa | 1.196±0.09Dc | 0.2811±0.004Bb |
|  | D5 | 11.47±1.42Cc | 32.52±0.23Ab | 54.48±1.7Ba | 0.00Aa | 0.4213±0.07BCb | 1.104±0.12Bc |

**Table S2a**: Beta diversity (%) in the gut microbiome of *Spodoptera frugiperda* larva after exposure to *Metarhizium anisopliae* strains ICIPE 7 and *Bacillus thuringiensis* strain Bt43 at 15 °C versus control calculated using the Jaccard dissimilarity index.

|  | ICIPE 7 (15 °C at Day 10) | Bt43 (15 °C at Day 10) | Control (15 °C at Day 0) | Control (15 °C at Day 10) |
| --- | --- | --- | --- | --- |
| ICIPE 7 (15 °C at D10) | - |  |  |  |
| Bt43 (15 °C at Day 10) | 74.74% | - |  |  |
| Control (15 °C at Day 0) | 95.73% | 96.65% | - |  |
| Control (15 °C at Day 10) | 86.27% | 88.17% | 98.75% | - |

**Table S2b:** Beta diversity (%) in the gut microbiome of *Spodoptera frugiperda* larva after exposure to *Metarhizium anisopliae* strain ICIPE 7 and *Bacillus thuringiensis* strain Bt43 at 20 °C versus control calculated using the Jaccard dissimilarity index.

|  | ICIPE 7 (20 °C at Day 10) | Bt43 (20 °C at Day 10) | Control (20 °C at Day 0) | Control (20 °C at Day 10) |
| --- | --- | --- | --- | --- |
| ICIPE 7 (20 °C at Day 10) | - |  |  |  |
| Bt43 (20 °C at Day 10) | 58.44% | - |  |  |
| Control (20 °C at Day 0) | 96.05% | 97.16% | - |  |
| Control (20 °C at Day 10) | 73.11% | 83.25% | 95.49% | - |

**Table S2c:** Beta diversity (%) in the gut microbiome of *Spodoptera frugiperda* larva after exposure to *Metarhizium anisopliae* strain ICIPE 7 and *Bacillus thuringiensis* strain Bt43 at 25 °C versus Control calculated using the Jaccard dissimilarity index.

|  | ICIPE 7 (25 °C at Day 10) | Bt43 (25 °C at Day 10) | Control (25 °C at Day 0) | Control (25 °C at Day 10) |
| --- | --- | --- | --- | --- |
| ICIPE 7 (25 °C at Day 10) | - |  |  |  |
| Bt43 (25 °C at Day 10) | 84.36% |  |  |  |
| Control (25 °C at Day 0) | 99.82% | 99.63% |  |  |
| Control (25 °C at Day 10) | 92.81% | 73.01% | 99.89% | - |
